# Peritoneal macrophages regulate distal wound healing via endocrine release of plasma fibronectin

**DOI:** 10.1172/JCI198632

**Published:** 2026-03-12

**Authors:** Lilian Salm, Simone N. Zwicky, Daniel Spari, Tural Yarahmadov, Marie Siwicki, Fernanda Vargas e Silva Castanheira, Jonas Zbinden, Deborah Stroka, Joel Zindel, Antoine Dufour, Paul Kubes, Guido Beldi

**Affiliations:** 1 Department of Visceral Surgery and Medicine, Inselspital, Bern University Hospital, University of Bern, Bern, Switzerland.; 2 Department for BioMedical Research, Visceral Surgery and Medicine, University of Bern, Bern, Switzerland.; 3 Calvin, Phoebe, and Joan Snyder Institute for Chronic Diseases and; 4 Department of Physiology and Pharmacology, Cumming School of Medicine and Calvin, University of Calgary, Calgary, Alberta, Canada.; 5 Department of Microbiology, Immunology and Parasitology, Federal University of São Paulo (UNIFESP/EPM), São Paulo, Brazil.; 6 Institute of Molecular Health Sciences, Department of Biology, Federal Institute of Technology (ETH) Zürich, Switzerland.; 7 McCaig Institute for Bone and Joint Health, University of Calgary, Calgary, Alberta, Canada.; 8 Department of Biomedical and Molecular Science, Queen’s University, Kingston, Ontario, Canada.

**Keywords:** Cell biology, Immunology, Fibronectin, Innate immunity, Macrophages

## Abstract

The peritoneal cavity contains a large population of GATA6-expressing large peritoneal macrophages (LPMs), known to support healing of intraabdominal organs. In this study, we aimed to explore their full sphere of influence by examining their ability to perform wound healing at distant sites outside the cavity. In a mouse model combining a remote skin injury with peritoneal stimulation we observed a significant acceleration of skin wound healing in response to LPM activation. Tracking GATA6-expressing LPMs, we demonstrated that LPMs do not migrate to distant wound sites following peritoneal activation. Using parabiosis experiments and administration of activated peritoneal contents indicated an important role of molecules secreted by LPMs in remote skin wound healing. More specifically, proteomic and transcriptomic analyses identified fibronectin as a key factor produced by activated LPMs. In fact, depletion of LPMs or genetic knockout of fibronectin in myeloid cells eliminated the enhanced healing effect. These findings highlight the endocrine function of LPMs in systemic tissue repair, challenging the traditional perspective of plasma fibronectin being exclusively liver derived. Our results suggest that LPMs, strategically positioned in the peritoneal cavity, serve as a source of circulating fibronectin, promoting matrix formation and accelerating wound healing at distant sites.

## Abstract 2

Peritoneal macrophages release plasma fibronectin that accelerates skin wound healing at distant sites, revealing their endocrine role in systemic tissue repair beyond the abdominal cavity.

## Introduction

Even 500 million years ago, primitive multicellular organisms like sea urchins could mount a centralized immune response (1–3). Macrophages within the coelomic cavity (coelomocytes) released cytokine- and clotting-like factors that could oppose distant breaches of the body wall (1, 4–6). In addition, large numbers of these coelomocytes within the coelomic cavity would quickly migrate to the injury site where they aggregated to seal and repair the wound (1, 7). Despite spectacular evolution of the immune system that includes the development of immune cells homing through blood vessels to peripheral sites of infection and sterile injury, a centralized innate immune hub has been preserved. In the case of infections, the liver and, more specifically, liver macrophages and hepatocytes, release cytokines and acute phase proteins into the blood stream to activate immunity and help fight pathogens (8). Despite the increasing complexity of anatomy, the presence of cavities that compartmentalize vital organs — while housing millions of macrophages — has been evolutionarily conserved from sea urchins to mammals (2, 5). Whether the centralized immune response that occurs during trauma or sterile injury that was so prevalent in primitive metazoan involved the peritoneal cavity remains unclear.

In mice, large peritoneal macrophages (LPMs) within the peritoneal cavity are characterized by the expression of the transcription factor GATA6, which partially induces their phenotypic identity (9–11). Comparable macrophage populations exist in humans; however, the role of GATA6 in human LPMs remains unclear due to poor detection of the transcriptions factor by antibodies and the extremely low or absent expression of its transcript (12, 13). Intravital imaging within the mouse peritoneal cavity demonstrated rapid recruitment of LPMs to a site of sterile peritoneal injury that initiated the process of intraabdominal healing. (14). However, whether these cells can be recruited to and heal distant sites is unclear. There is evidence to support this notion. Following various systemic stimuli, LPMs have been observed to disappear from the peritoneal cavity, with one possible explanation being their migration to distal organs (5, 15–17). In these distant organs, LPMs can survive and adapt their new environment; for example, when transplanted from the peritoneal cavity into the lung airways they not only survived but adapted to the new environment, including the downregulation of *Gata6*; the linage tracing marker for these cells, and the upregulation of alveolar macrophage-specific marker (18). One study attempted to track LPMs from the peritoneum and reported that they could migrate into distal sites of injury (19). However, this study was confounded by the fact that LPMs rapidly downregulate *Gata6* once they leave the confines of the peritoneal cavity and, therefore, conclusions are impossible without an effective macrophage lineage tracer (19).

Alternatively, LPMs could function like liver macrophages and, upon distant stimulation, they could release bioactive healing factors systemically (20, 21). Recent work has shown the tremendous capacity that LPMs have for extracellular matrix (ECM) protein deposition (22). In cases of abdominal surgery, LPMs mediate the growth of intraabdominal scar tissue called peritoneal adhesions through deposition of ECM proteins (23–25). With this in mind, and the fact that the peritoneal cavity has a very robust lymphatic drainage system allowing intraperitoneal injection of molecules as large as antibodies to rapidly disseminate into blood (26–28), we hypothesized that the sphere of influence of LPMs extends beyond the peritoneal cavity to sites lacking direct contact with these cells. We took a systematic approach and first developed a simple model of remote skin injury with and without stimulation of LPMs within the peritoneal cavity and noted a robust improvement in healing when LPMs were activated. Using a *Gata6* tracer mouse to track LPMs throughout the body, we were unable to find any evidence that LPMs trafficked to injuries outside the peritoneal cavity. However, transcriptomic and proteomic analyses showed that LPMs produce and release many bioactive molecules, including complement factors and acute-phase proteins like fibronectin (FN; ref. 22). These LPM-derived soluble factors were secreted at concentrations high enough to promote a distant skin wound healing response. Complement proteins and FN emerged as the most prominent proteins, but only depletion of FN specifically from myeloid cells abolished the enhanced remote wound healing response. The data suggest that LPMs strategically positioned in the permeable peritoneal cavity can produce sufficient FN to significantly affect distal repair at sites such as skin, which is not in direct contact with these critical repair macrophages.

## Results

### Stimulation of the peritoneal cavity accelerates remote skin wound healing.

**Figure 1:**
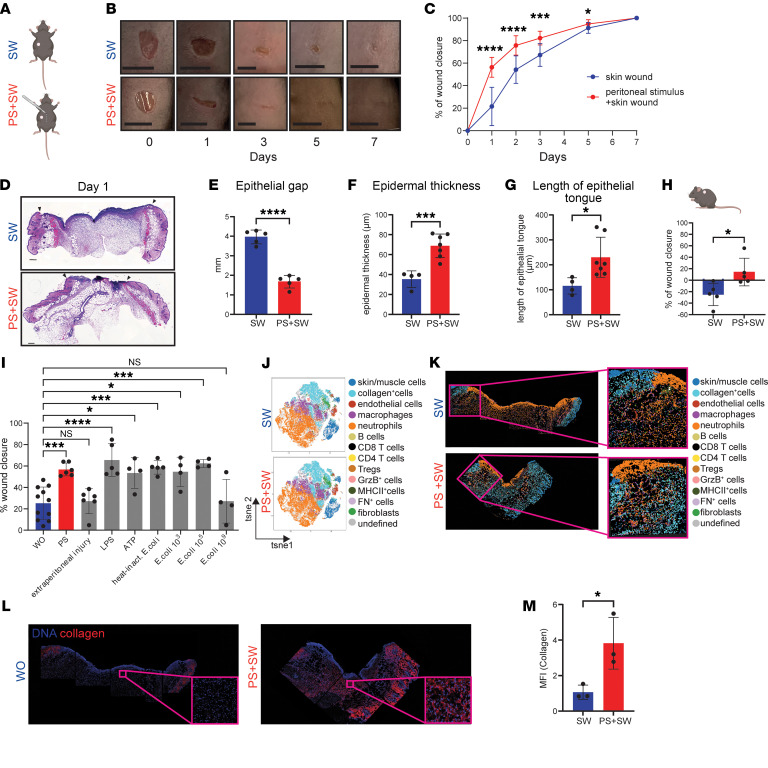
Peritoneal stimulus accelerates skin wound healing. (**A**) Illustration of mouse model with punch excision (diameter 4 mm) in the wound-only (SW) group. For peritoneal stimulus (PS), median laparotomy was performed at same time as punch excision (**B**) Representative photographs of wound closure at days 0, 1, 3, 5, and 7; scale bar: 500 μm (**C**) Quantification of percentage wound closure rate (mean ± SEM) at days 1, 2, 3, 5, and 7 after injury using digital calipers. Percent wound closure calculated [(Ai-Af)/Ai], Ai represents initial wound area and Af represents final wound area. *n* = 12 (SW), *n* = 13 (PS + SW) for each timepoint (*N* = 3). (**D**) Representative H&E slides (**E**) and measurement of epithelial gap, day 1; black arrow, wound margin; scale bar: 200μm. *n* = 5 (both groups), *N* = 2, *P* ≤ 0.0001 (**D** and **E**). (**F**) Quantification of epidermal thickness, day 1, *n* = 4 (SW), *n* = 6 (PS + SW), *N* = 2, *P* = 0.0008. (**G**) Quantification length of epithelial tongue, day 1, *n* = 4 (SW), *n* = 6 (PS + SW), *N* = 2, *P* = 0.0252. (**H**) Quantification of percentage wound closure at dorsum, day 1, *n* = 5 each group, *N* = 2, *P* = 0.0231. (**I**) Quantification of percentage wound closure, day 1. SW (*n =* 11), PS + SW (*n =* 6), extraperitoneal injury (*n =* 6), LPS (*n =* 5), ATP (*n =* 4), heat-inactivated *E*. *coli* (*n =* 5), *E*. *coli* 10^3^ (*n =* 4), *E*. *coli* 10^5^(*n =* 4), *E*. *coli* 10^9^(*n =* 4), *N =* 2, SW + PBS / PS + SW + PBS: *P =* 0.0007, SW + PBS / LPS: *P* < 0.0001, SW + PBS / ATP: *P =* 0.0156, SW + PBS / heat-inact. *E*. *coli*: *P =* 0.0007, SW + PBS / *E*. *coli* 10^3^: *P =* 0.0105, SW + PBS / *E*. *coli* 10^5^: *P =* 0.0005, *F* = 9.846 (**K**) Representative IMC images of skin wound 24 hours, SW or PS + SW, SW (*n =* 3), PS + SW (*n =* 3), *N =* 1 (**J**) t-SNE plot showing 14 distinct cell types in the skin wounds, 24 hours postoperative (**L**) Representative IMC images, DNA (blue), collagen (red) in skin wounds (**M**) Quantification of collagen (MFI), *P =* 0.0343 unpaired t test. Unless otherwise indicated, images and data denote single mice from one representative experiment; independent experiments yielded similar results (representative of N ≥ 2 [*N =* 1 IMC]). **K** and **L** are generated from same IMC tissue section.*P < 0.05, **P < 0.01, ***P < 0.001; Student’s *t* test (**C**, **D**, **E**, **F**, **G**, **H**, **M**); 1-way ANOVA with Tukey`s post hoc test (**I**) **A** and mouse scheme of **H** was created using BioRender.

To evaluate whether LPMs have systemic wound healing capabilities, we developed a mouse model of remote wound healing. A 4-mm excisional skin wound (SW) was applied on the right flank, and simultaneously, a peritoneal stimulus (PS+SW) was induced by a simple laparotomy (Figure 1A). The control group consisted of mice with a skin wound only (SW) (Figure 1A). Mice subjected to PS+SW exhibited significantly accelerated wound healing at 24 hours, with approximately 60% (mean ± SEM) wound closure compared with approximately 20% in the SW control group (Figure 1, B and C). Epidermal thickness at the wound edges was increased, epithelial tongue length was longer, and the epithelial gap was reduced in PS+SW compared with the SW control group, indicating enhanced reepithelialization upon peritoneal stimulus (Figure 1, D–G, and Supplemental Figure 1A; supplemental material available online with this article; https://doi.org/10.1172/JCI198632DS1). This enhanced wound healing response persisted until postoperative day 5 (Figure 1, B and C). Consistent with our main findings, PS+SW also accelerated closure of dorsal skin wounds, rather than being restricted to flank wounds, demonstrating that the effect of peritoneal stimulus on wound healing is generalizable across different skin sites (Figure 1H). In contrast, a ventral incision that did not penetrate the peritoneal cavity (extraperitoneal injury) did not accelerate remote skin wound closure (Figure 1I), indicating that peritoneal perturbation is essential for the observed effect. Administration of LPS, ATP, heat-inactivated *E*. *coli,* and even live *E*. *coli* into the peritoneal cavity was sufficient to improve the remote skin wound closure (Figure 1I). We first examined the immune cell populations by flow cytometry at the skin wound site in PS+SW versus SW. No difference in absolute numbers of platelets, monocytes, macrophages, or neutrophils were found at the injury site at 2 and 24 hours after injury (Supplemental Figure 1, B–D). Opening the peritoneum was not sufficient to alter the numbers of immune cells in the blood between conditions (Supplemental Figure 2, A and B). Next, skin wound composition upon peritoneal stimulus was assessed by imaging mass cytometry (IMC) using a 34-target antibody panel (Figure 1, J–M, and Supplemental Figure 3, A and B). At 24 hours, fractions of fibroblasts (vimentin^+^ and αSMA^+^), endothelial-cells (EC), collagen^+^-cells, keratinocytes, macrophages, neutrophils, T cells (CD4, CD8 and Tregs), B cells, and MHCII^+^-cells did not differ between conditions (Figure 1, J and K, and Supplemental Figure 3, A and B). However, we found increased ECM protein, such as collagens, in the skin wounds of mice subjected to peritoneal stimulus as an indicator of enhanced tissue repair (Figure 1, L and M) (29).

### Large peritoneal macrophages mediate remote skin wound healing.

**Figure 2:**
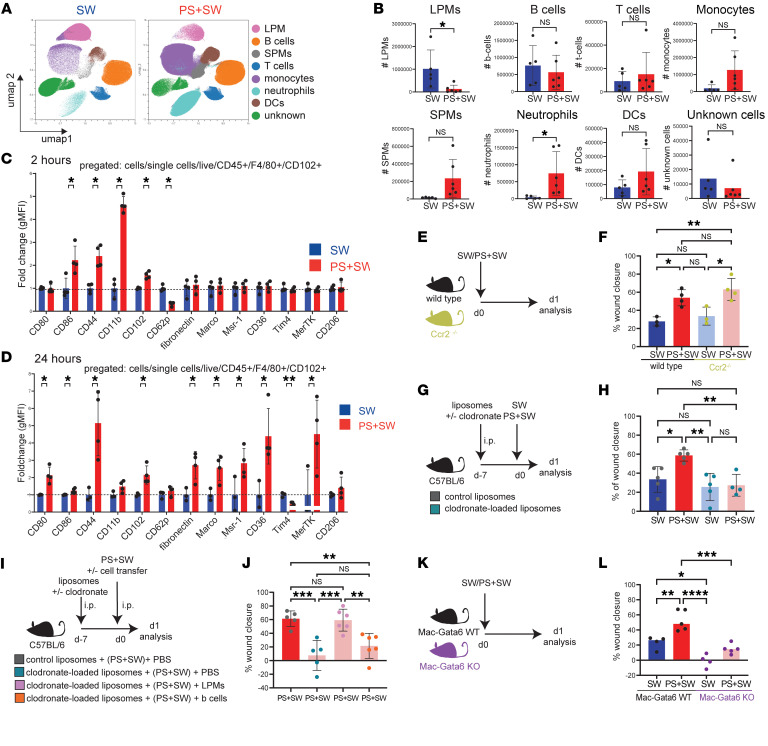
Large peritoneal macrophage B cells mediate remote skin healing. (**A**) Flow cytometry on peritoneal fluid. UMAP-plot (*n =* 6 animals each condition) of cellular changes after SW and PS + SW after 24 hours. Cells were pregated on size, single cells, and viability. *N =* 3. (**B**) Quantification of LPMs, B cells, T cells, monocytes, SPMs, neutrophils, DCs, unknown cells (SW: *n =* 5, PS + SW: *n =* 6), *N =* 3. LPMs: *P =* 0.0318, B cells: 0.5666, T cells: *P =* 0.5359, monocytes: 0.0671, SPMs: 0.0516, neutrophils: *P =* 0.0384, DCs: 0.1797, unknown cells: *P =* 04108 (**C**) Fold-change MFI of macrophage markers expressed on LPMs (SW: *n =* 4, PS: *n =* 4) at 2 hours, *N =* 2. LPMs are pregated as single, live, CD45^+^, F4/80^+^, CD102^+^. CD86: *P =* 0.017836, CD44: 0.001569, CD11b: *P =* 0.000007, CD102: *P =* 0.000367, (**D**) Fold-change MFI of macrophage markers expressed on LPMs (SW: *n =* 4, PS: *n =* 4) after 24 hours. *N =* 2. LPMs are pregated as single, live, CD45^+^, F4/80^+^, CD102^+^. CD80: *P =* 0.009962, CD44: 0.014507, CD102: *P =* 0.015040, fibronectin: *P =* 0.019421, Marco: *P =* 0.019421, Msr-1: *P =* 0.047060, CD36: *P =* 0.021312, Tim4: *P =* 0.002588, MerTK: *P =* 0.048907, (**E**) Illustration of experimental approach. (**F**) Quantification of percentage wound closure, day 1. SW(WT): *n =* 3, PS + SW(WT): *n =* 4, SW(*Ccr2* ^–/–^): *n =* 3, PS + SW(*Ccr2* ^–/–^): *n =* 4), *N =* 2. SW(WT) / PS + SW(WT): *P =* 0.0236, SW(WT) / SW(*Ccr2* ^–/–^): *P =* 0.8804, SW(WT) / PS + SW(*Ccr2* ^–/–^): *P =* 0.0034, PS + SW(WT) / SW(*Ccr2* ^–/–^): *P =* 0.5521, SW(*Ccr2* ^–/–^) / PS + SW(*Ccr2* ^–/–^): *P =* 0.0113. (**G**) Illustration of experimental approach. (**H**) Quantification of % wound closure, day 1.(SW + PBS liposomes: *n =* 5, PS + SW + PBS liposomes: *n =* 5, SW + Clodronate liposomes: *n =* 5, SW + PBS liposomes: *n =* 4), *N =* 3. SW(PBS liposomes) / PS + SW(PBS liposomes): *P =* 0.0194, PS + SW(PBS liposomes) / SW(Clodronate liposomes): *P =* 0.0024, PS + SW(PBS liposomes) / PS + SW(Clodronate liposomes): *P =* 0.0062, (**I**) Illustration of experimental approach. (**J**) Quantification of percentage wound closure, day 1. (PBS liposomes + (PS + SW) + PBS: *n =* 5, clodronate liposomes + (PS + SW) + PBS: *n =* 5, clodronate liposomes + (PS + SW) + LPMs *n =* 6, clodronate liposomes + PS + B cells: *n =* 6), *N =* 2. PS + SW (PBS liposomes) / PS + SW(Clodronate liposomes): *P =* 0.0006, PS + SW(PBS liposomes) / PS + SW(Clodronate liposomes + B cells): *P =* 0.0067, PS + SW (Clodronate liposomes) / PS + SW(Clodronate liposomes + LPMs): *P =* 0.0006, PS + SW (Clodronate liposomes + LPMs) / PS + SW(Clodronate liposomes + B cells): *P =* 0.0070, (**K**) Illustration of experimental approach. (**L**) Quantification of percentage wound closure, day 1. (SW(WT): *n =* 4, PS + SW(WT)): *n =* 5, SW(Mac Gata6 KO):*n =* 4, PS + SW(Mac Gata6 KO): *n =* 5), *N =* 2, SW(WT) /PS + SW(Mac Gata6 WT): *P =* 0.0029, SW(WT) / SW (Mac Gata6 KO) *P =* 0.0182, PS + SW(WT) / SW(Mac Gata6 KO): *P* < 0.0001, PS+SW(WT)/PS+SW(Mac Gata6 KO Cre+): *P =* 0.0002. (**B**) Students *t* test (**C**, **D**) multiple unpaired 2-tailed *t* tests**,** (**F**, **H**, **J**, **L**) 1-way ANOVA with Tukey`s post hoc test. Each data point represents an individual mouse from one representative experiment. Independent experiments yielded similar results and are representative of N ≥ 2 experiments.

To determine alterations in the peritoneum leading to accelerated remote wound healing, we analyzed changes in the cell populations within the peritoneal cavity. At 24 hours after peritoneal stimulation, only LPM numbers, as measured by flow cytometry, decreased in the peritoneal cavity, as expected (15, 16, 30, 31) (Figure 2, A and B, and Supplemental Figure 4A). However, neutrophils, monocytes, and small peritoneal macrophages (SPMs) increased in numbers, while B cells- and T cells did not change (Figure 2, A and B). Multiparametric spectral flow cytometry was used to further profile LPMs. LPMs were isolated from PS+SW and SW mice and gated based on their expression of F4/80^high^CD102^+^ (Gating strategy, Supplemental Figure 4B). Opening the peritoneal cavity rapidly activated LPMs within 2 hours, including the upregulation of adhesion molecules (CD11b, CD102, CD44) and the activation marker CD86, alongside a reduction in P-Selectin levels (CD62p) (Figure 2C). At 24 hours, increase in expression of the activation markers (CD80, CD86), scavenger receptors (Marco, Msr-1, and CD36), and the efferocytosis receptor MerTK were observed (Figure 2D). Conversely, the expression of the efferocytosis receptor Tim4 decreased (Figure 2D). These findings suggest a shift in the phenotype of the remaining LPMs from a migratory state towards a more reparative and scavenging phenotype.

To determine whether monocytes contribute to the accelerated wound healing phenotype upon PS, we examined *Ccr2* ^–/–^ mice, in which monocyte recruitment is impaired (32). PS still accelerated remote skin wound healing in *Ccr2* ^–/–^ mice (Figure 2, E and F).

Intraperitoneal injection of small concentrations of clodronate-loaded liposomes 7 days prior to PS+SW or SW challenge alone was used to reduce LPM numbers but not macrophages in other organs such as skin or liver (Supplemental Figure 4C) (33). Moreover, the recruitment of neutrophils, monocytes, and SPMs was not altered upon the treatment with clodronate-loaded liposomes (Supplemental Figure 4, D and E). This depletion of LPMs resulted in abrogation of accelerated remote wound healing, suggesting that peritoneal macrophages play a critical role in enhancing remote skin wound healing (Figure 2, G and H, and Supplemental Figure 4, F–H). There was no difference in wound healing in the SW group with or without macrophage depletion, supporting that LPMs require a peritoneal stimulus to enhance remote wound healing (Figure 2, G and H). Adoptive transfer of LPMs but not B cells (second largest cellular compartment of the peritoneal cavity) at 7 days after LPM depletion was sufficient to restore the improved remote skin wound healing after peritoneal stimulation (Figure 2, I and J). Because chemical depletion using clodronate-loaded liposomes can have some untoward effects (34) we also assessed the remote skin wound healing response in *LyzM^cre^ Gata6^fl/fl^* mice (Mac-Gata6 KO), which specifically lack GATA6^+^LPMs (9–11). PS+SW in Mac-Gata6–KO mice did not induce a significant remote skin wound healing response (Figure 2, K and L). In the Mac-Gata6-KO mice, the recruitment of neutrophils, monocytes, and SPMs upon peritoneal stimulus was not altered (Supplemental Figure 5, A and B). Together, these results show that LPMs are activated upon peritoneal stimulation and are required for accelerated remote skin wound healing.

### Accelerated remote skin wound healing is not due to recruitment of LPMs.

**Figure 3:**
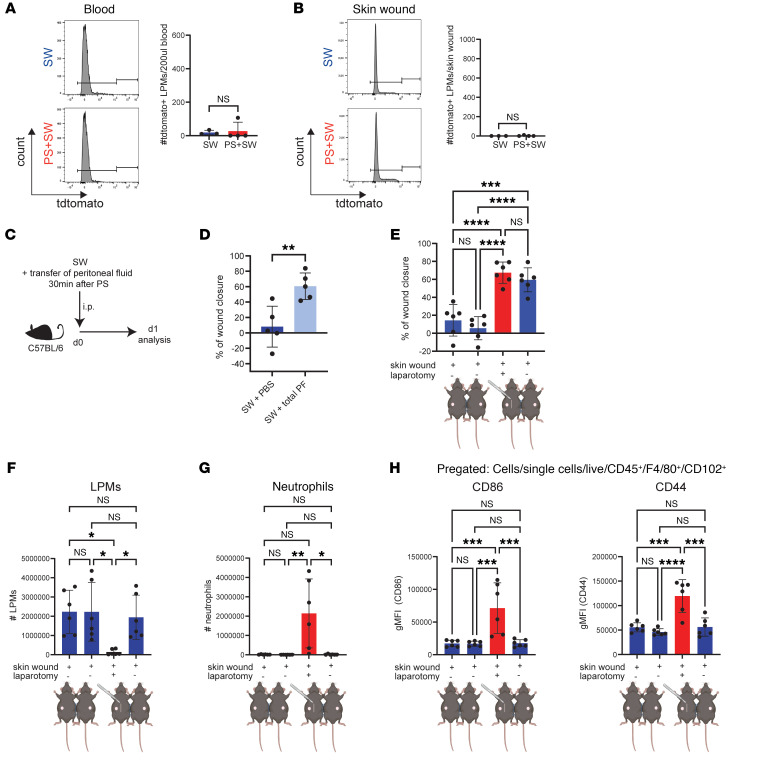
LPM-derived soluble factor promote skin wound healing following peritoneal stimulus. (**A**) Representative flow cytometry histogram of tdtomato^+^ cells in the blood from SW + PS mice. Cells were pregated as live, single, CD45^+^ cells. Quantification of of tdtomato^+^ cells in the blood at day 1, (SW: *n =* 3, PS: *n =* 4), *N =* 3 *P =* 0.8176. (**B**) Representative flow cytometry histogram of tdtomato^+^ cells in the skin wound from SW and PS mice. Cells were pregated as live, single, CD45^+^ cells. Quantification of of tdtomato^+^ cells in the skin wound at day 1, (SW: *n =* 3, PS: *n =* 4), *N =* 3, *P =* 0.4366. (**C**) Illustration of experimental approach (**D**) Quantification of percentage wound closure, day 1. (SW + PBS: *n =* 5, SW + total PF: *n =* 5), *N =* 2 (SW + PBS) / (SW + total PF): *P =* 0.0060, *N =* 2 (**E**) Quantification of percentage wound closure, day 1, *n =* 6 in each group, *N =* 2, Column A / Column B: *P =* 0.7110, Column A / Column C: *P <* 0.0001, Column A / Column D: *P =* 0.0001, Column B / Column C: *P <* 0.0001, Column B / Column D: *P <* 0.0001, Column C / Column D: *P =* 0.7652. (**F**) Quantification of LPMs, *n =* 6 in each group, *N =* 2, Column A / Column B: *P >* 0.9999, Column A / Column C: *P =* 0.0132, Column A / Column D: *P >* 0.9999, Column B / Column C: *P =* 0.0132, Column B / Column D: *P >* 0.9999, Column C / Column D: *P =* 0.0423. (**G**) Quantification of neutrophils, *n =* 6 each group, *N =* 2, Column A / Column B: *P =* 0.4751, Column A / Column C: *P =* 0.3301, Column A / Column D *P >* 0.9999, Column B / Column C: *P =* 0.0014, Column B / Column D: *P >* 0.9999, Column C / Column D: *P =* 0.0132. (**H**) geometric MFI (CD86) on LPMs, cells pregated live, single, CD45^+^ F4/80^hi^ CD102^+^ cells. *n =* 6 in each group, *N =* 2. CD86: Column A / Column B: *P >* 0.9999, Column A / Column C: *P =* 0.0007, Column A / Column D: *P >* 0.9999, Column B / Column C: *P =* 0.0006, Column B / Column D: *P >* 0.9999, Column C / Column D: *P =* 0.0007, CD44: Column A / Column B: *P =* 0.8451, Column A / Column C: *P =* 0.0001, Column A / Column D: *P >* 0.9999, Column B / Column C: *P <* 0.0001, Column B / Column D: *P =* 0.8146, Column C / Column D: *P =* 0.0001. Each data point represents an individual mouse from one representative experiment. Independent experiments yielded similar results and are representative of N ≥ 2 experiments. Student’s *t* test (**A**, **B**, **D**)**,** 1-way ANOVA with Tukey’s post hoc test (**E**, **H**), Kruskal-Wallis test (**F**, **G**). Mouse scheme of **E**–**H** was created using BioRender.

We hypothesized that LPMs migrate from the peritoneal cavity to the skin wound. LPMs are the only immune cells to express GATA6; however, many parenchymal cells also express GATA6, particularly during development (9, 10, 35). LPMs rapidly down regulate GATA6 when migrating into visceral organs or a tumor environment that lacks retinoic acid (36, 37), making it difficult to track them outside the peritoneal cavity. To overcome this limitation, we created a transgenic mouse strain by crossing a Lysozyme M–driven Cre recombinase strain with a Gata6-driven tamoxifen-inducible Flippase, followed by crossing with an Ai65 reporter strain containing both Cre recombinase- and Flippase-sensitive stop sequences upstream of the tdTomato gene: *LyzMCre GATA6FlipER Ai65(RCFL-tdT)*.This enables tamoxifen to permanently induce red fluorescence exclusively in GATA6^+^ cavity macrophages, referred to as GATA6-RED LPMs. We could not detect any GATA6 RED LPMs in blood or at the skin wound after peritoneal stimulation (Figure 3, A and B). These data indicate that LPMs were not migrating beyond these serosal cavities to the skin wound in our model of remote wound healing.

### Accelerated remote skin wound healing is mediated by a soluble factor.

We hypothesized that the LPMs were releasing factors that accelerate remote skin wound healing. Indeed, adoptive transfer of peritoneal fluid, which was obtained 30 minutes after peritoneal stimulation from a donor mouse, was sufficient to induce accelerated remote skin wound healing at day 1 in a mouse exposed to a skin wound (Figure 3, C and D). Next, a parabiosis experiment was performed wherein the peritoneum of one mouse of the parabiotic pair was stimulated and the other mouse received a skin wound only (Figure 3E). We observed accelerated skin wound healing in the partner mouse (Figure 3E). For completeness, the parabiosis mouse that received peritoneal stimulation was also able to heal faster (Figure 3E). LPM activation (CD86, CD44), decrease in LPM number, and peritoneal neutrophil infiltration remained a local effect in the mouse that received the peritoneal stimulus (Figure 3, F–H). These effects were not present in the parabiotic partner, supporting the concept of releasing of a factor into the blood circulation for improved skin wound healing (Figure 3, F–H).

### LPM-derived complement does not accelerate remote skin wound healing.

**Figure 4:**
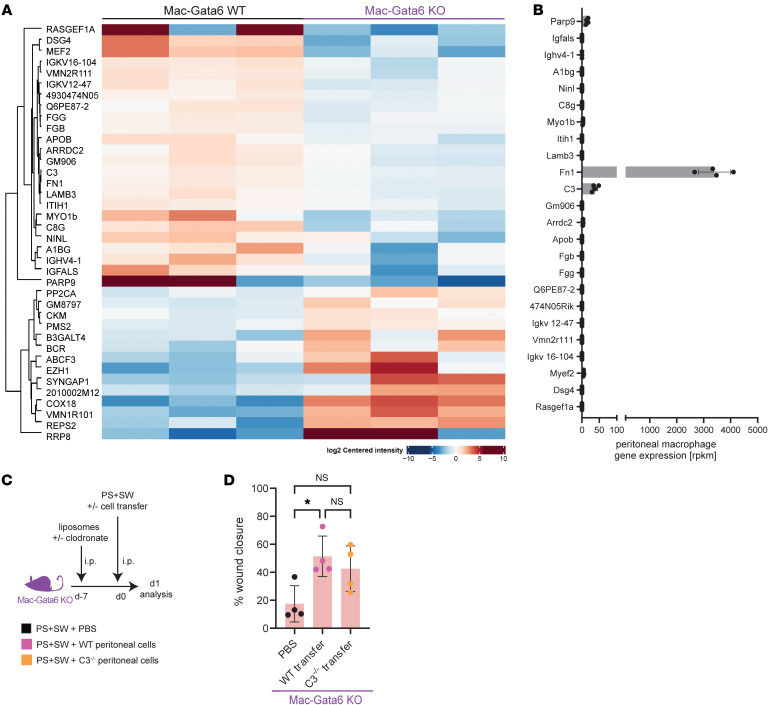
LPM-derived complement does not accelerate remote skin wound healing. (**A**) Proteins identified by proteomics analysis are represented as heatmap. 2-sided analysis was performed, and it was adjusted for multiple comparison. (**B**) RNA-seq gene expression levels of proteins identified by proteomics. (**C**) Illustration of experimental approach (**D**) Quantification of percentage wound closure, day 1. (PBS: *n =* 4, WT transfer: *n =* 4, C3 transfer: *n =* 4), *N =* 2 PBS / WT transfer: *P =* 0.0235, PBS / C3^–/–^ transfer: *P =* 0.6845, (1-way ANOVA with Tukey`s post hoc test), *N =* 2. Each data point represents an individual mouse from one representative experiment. Independent experiments yielded similar results and are representative of N ≥ 2 experiments.

To investigate which factors released by LPMs are involved in the remote skin wound healing phenotype, we conducted a proteomic analysis using liquid chromatography and tandem mass spectrometry (LC-MS/MS) on the peritoneal supernatant collected 30 minutes after peritoneal stimulation from Mac-Gata6–KO and Mac-Gata6–WT mice. We identified 24 proteins that were significantly increased in the peritoneal fluid in the Mac-Gata6–WT mice compared with the Mac-Gata6–KO mice. (Figure 4A). By cross referencing the proteomic results with the analysis of recently published transcriptomic profiles of isolated peritoneal macrophages (Figure 4B) (36), we found that Fibronectin *(Fn1)* and Complement 3 *(C3)* are the most abundant molecules expressed and produced by LPMs (Figure 4, A and B). First, the effect of C3 was assessed, given the relevance of complement pathways as important factors for repair (38, 39). Adoptive transfer of WT LPMs into Mac-Gata6–KO mice induced an accelerated remote skin healing phenotype that was also observed in Mac-Gata6–KO mice receiving C3^–/–^ LPMs, suggesting that complement was not involved in remote skin wound healing (Figure 4, C and D).

### FN released by LPM reaches remote skin wounds via the blood stream.

**Figure 5:**
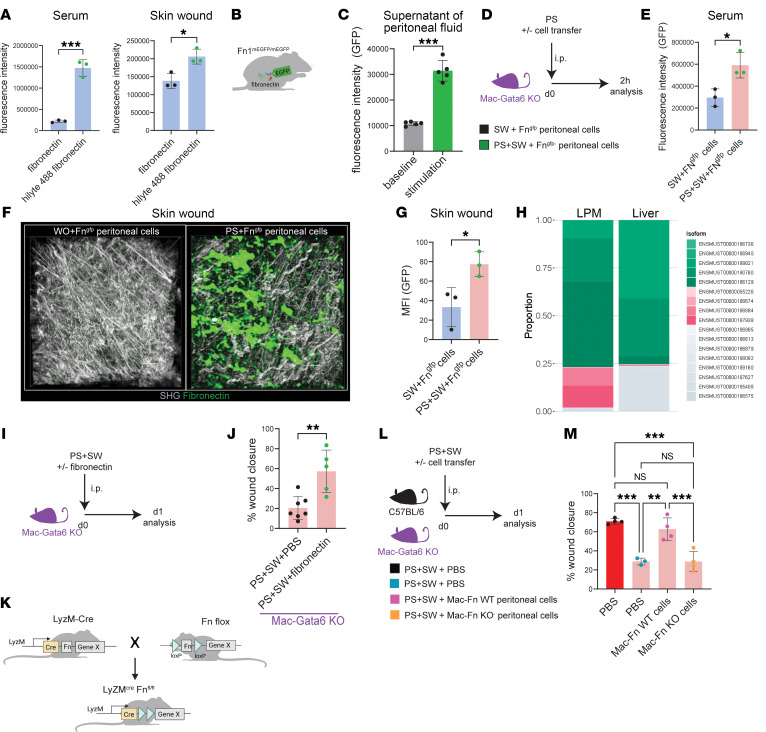
Fibronectin released by LPM reaches remote skin wounds via blood stream. (**A**) Quantification of fluorescence intensity, serum: (fibronectin: *n =* 3, hilyte 488 fibronectin: *n =* 3), *N =* 3, *P =* 0.0004, skin wound: *P =* 0.0167. (**B**) Fn^mEGFP/mEGFP^ mouse scheme (**C**) Quantification of fluorescence intensity (GFP) after stimulation of the peritoneal fluid with ATP + Ca2^+^. (baseline: *n =* 5, stimulation: *n =* 5), *N =* 3, *P =* 0.0002. (**D**) Illustration of experimental approach. (**E**) Quantification of fluorescence intensity (GFP) in the serum. SW+FN^gfp^ cells / PS+SW+FN^gfp^ cells: (SW+FN^gfp/gfp^ cells: *n =* 3, PS+SW+FN^gfp/gfp^ cells: *n =* 3), *N =* 2. *P =* 0.0217. (**F**) Skin wound IVM 2 hours after surgery. Images are representative of quantification shown in **G** (**G**) Quantification MFI in the skin wound. (SW+FN^gfp/gfp^ cells: *n =* 3, PS+SW+FN^gfp/gfp^ cells: *n =* 3), *N =* 2. SW+FN^gfp^ cells/PS+ SW+FN^gfp^ cells: *P =* 0.0328, *N =* 2. (**H**) fibronectin isoform composition from bulk RNA-seq of whole liver tissue and sorted LPMs. (**I**) Illustration of experimental approach (**J**) Quantification of percentage wound closure, day 1. (PBS: *n =* 7, fibronectin: *n =* 5), *N =* 3, PS+SW+PBS / PS+SW+FN: *P =* 0.0031, *N =* 2 (**K**) Generation of LyzM^cre^Fn^fl/fl^ mice (**L**) Illustration of experimental approach (**M**) Quantification of percentage wound closure, day 1. (WT+PBS: *n =* 4, KO+PBS *n =* 3, KO+WT cells: *n =* 4, KO+FN KO cells: *n =* 4), *N =* 2. Mac-Gata6 WT+PBS / Mac-Gata6 KO: *P =* 0.0002, Mac-Gata6 WT+PBS / Mac-Gata6 KO+Mac-FN KO cells: 0 = 0.0001, Mac-Gata6 KO+PBS / Mac-Gata6KO+Mac Fn WT cells: *P =* 0.0014, Mac-Gata6 KO+ Mac-FN WT cells/Mac Gata6 KO+ Mac-Fn KO cells: *P =* 0.0008. Images and each data point represent an individual mouse from one representative experiment. Independent experimental repetitions yielded similar results and are representative of N ≥ 2 experiments. Student’s *t* test (**A**, **E**, **G**, **J**); 1-way ANOVA with Tukey’s post hoc test (**M**); paired *t* test (**C**). Panels **B** and **K** were created using BioRender.

As a proof-of-principle experiment, we verified whether FN injected into the cavity would home to a skin wound. Therefore, fluorescent-tagged FN was injected into the peritoneal cavity of mice, followed by an induction of a skin wound. Within 2 hours, fluorescent signal was detected in both the serum and skin wound (Figure 5A). Interrogation of publicly available single-cell RNA-seq data (13) further demonstrated that GATA6^+^ LPMS highly express FN, whereas CCR2^+^ monocytes express low levels of FN, supporting LPMs as the predominant FN-producing population after peritoneal stimulation (Supplemental Figure 6, A and B). To confirm that peritoneal cells were the source of the FN, we next used a reporter mouse expressing FN tagged with mEGFP. (Fn1^mEGFP^ mice) (Figure 5B) (40). We detected increased fluorescent FN in the supernatant after stimulation of peritoneal cells with ATP and Ca^2+^ in vitro (Figure 5C) (14). Adoptive transfer of Fn1^mEGFP^ LPMs into the peritoneal cavity resulted in detectable fluorescent FN in the serum within 2 hours of peritoneal stimulation (Figure 5, D and E). No fluorescence was detected in the serum of mice that did not receive the peritoneal stimulus (Figure 5, D and E). Intravital imaging revealed fluorescent FN signal at the remote skin wound site upon adoptive transfer of Fn1^mEGFP^ cells and peritoneal stimulation (Figure 5, F and G). At least 20 different FN isoforms in humans are described (41). Alternative splicing of a single pre-mRNA determines the isoforms that can be broadly categorized in soluble “plasma-” and insoluble “cellular-” FN. Plasma-FN lacks the ED-A and ED-B domains and is considered to be mainly derived from the liver. Conversely, cellular-FN contains variable amounts of these 2 domains and is synthesized by various cells locally in tissues (42–44). Our data demonstrate that peritoneal FN reaches skin wounds via the bloodstream. This prompted us to investigate whether, in addition to the liver, LPMs can also secrete soluble plasma-FN, challenging the established dogma in the literature. Therefore, a differential transcript analysis of a published bulk sequencing data of a peritoneal macrophage set was performed (36). We identified 7 different protein-coding isoforms transcribed by LPMs (Table 1). Interestingly, transcripts, which are classified as plasma-FN, are highly expressed in LPMs (Figure 5H). This indicates that soluble plasma-FN is not only produced in the liver. Approximately 75% of the FN transcripts from LPMs are plasma-FN (green), a proportion that mirrors the findings from liver bulk sequencing (Figure 5H) (45). The main difference between hepatic and peritoneal plasma-FN is the ENSMUST000000186129 isoform that dominates in LPM and the ENSMUST000000189821 isoform that dominates in the liver (Figure 5H).

Next, we asked whether injection of FN could affect remote skin wound healing to mimic a potential therapeutic application. The ip injection of FN was sufficient to induce an accelerated remote skin wound healing phenotype following PS+SW in Mac-Gata6–KO mice and thereby successfully rescued the impaired phenotype observed in these mice (Figure 5, I and J). The liver is traditionally viewed as the main source of FN in our body. To test whether LPM-derived FN mediates the phenotype observed, we next generated a myeloid-cell–specific FN knockout mouse (Mac-FN KO) (Figure 5K). Adoptive transfer of peritoneal cells from Mac-FN–WT donors but not from Mac-FN–KO donors accelerated remote skin wound closure after PS+SW in Mac-Gata6–KO recipients (Figure 5, L and M). These data suggest that FN released by peritoneal macrophages may exert a systemic effect, contributing to its role in mediating skin wound healing.

These results demonstrate that plasma FN is released by LPM from the peritoneal cavity upon stimulation and reaches the distant wound to accelerate skin wound healing responses.

### Human peritoneal macrophages also express FN needed for healing.

**Figure 6:**
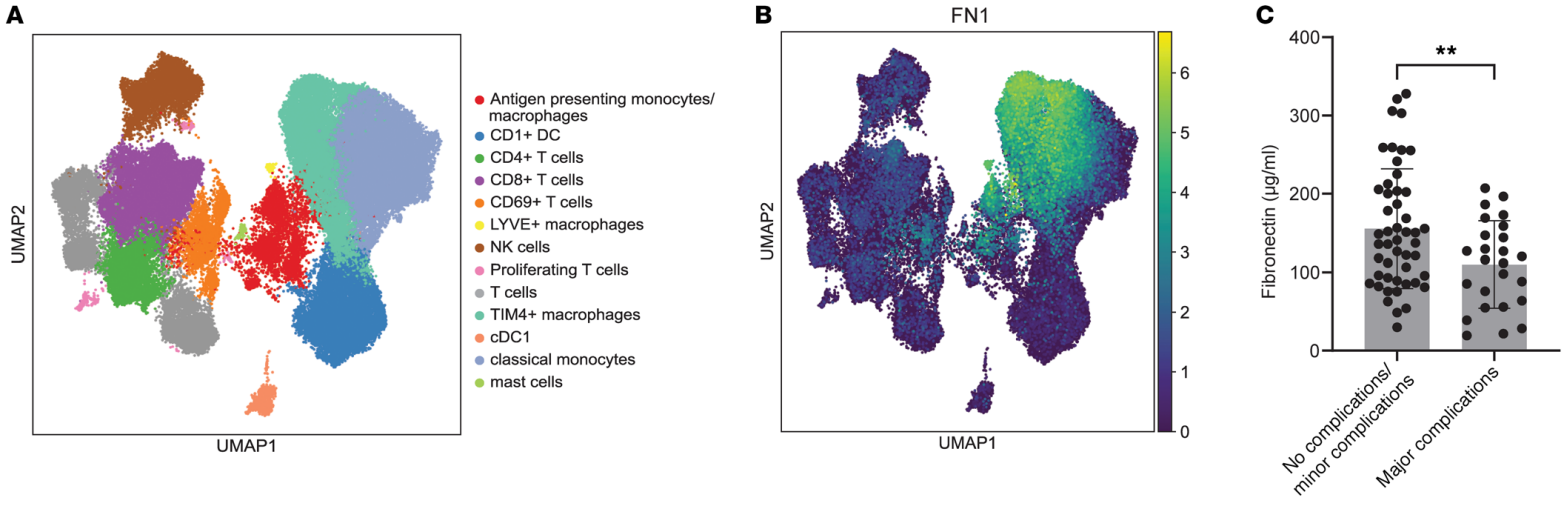
Skin wound composition upon peritoneal stimulus. (**A** and **B**) **A** and **B** were generated using publicly available single cell sequencing data from ref. 13. (**A**) UMAP-plot of human peritoneal cells and (**B**) Fibronectin expression within these cells. (**C**) Quantification of preoperative serum fibronectin and its association with postoperative minor and major complications: *n =* 72, *N =* 1, *P =* 0.0096, (Student’s *t* test).

We next reanalyzed a recently published single-cell RNA-seq dataset (13) of healthy human peritoneal cells and found that Tim4^+^ large-cavity macrophages (equivalent to mouse LPMs) exhibited the highest levels of FN expression (Figure 6, A and B, and Supplemental Figure 7A). We next assessed levels of serum FN in patients undergoing major abdominal surgery and found a significant correlation with morbidity and mortality. Low preoperative FN serum levels were associated with major postoperative complications such as pancreatic fistula, anastomotic leakage postoperative hemorrhage, or intraabdominal abscess (Clavien-Dindo grade >3) (Figure 6C) (46). While these results are only associative, they do support the hypothesis that peritoneal stimulation leads to FN release in humans and is linked to improved outcome. This also raises the possibility that FN may serve as a potential biomarker for predicting surgical outcomes.

## Discussion

In this study, we have identified that the peritoneal cavity and, more specifically, the LPMs, have the capacity to perform endocrine functions by releasing sufficient amounts of soluble FN that enables long-range effects on wound healing. While LPMs were previously recognized for their role in local healing following injury to the abdominal wall or visceral organs, we now demonstrate that they can also mediate healing of a remote skin wound, which lacks both a cavity and macrophages resembling LPMs (14, 33, 36, 47). Our original hypothesis was that LPMs left the peritoneal cavity and migrated to the injury site, but existing tools were insufficient to test this premise. Thus, using our lineage-tracing mouse model, we did not find any evidence of LPMs migrating beyond the confines of the peritoneal cavity in our model. We therefore refined our hypothesis to suggest that the LPM healing effects were through release of a soluble factor into the extremely well drained peritoneal cavity, often used for systemic delivery of various molecules. Indeed, using myeloid-cell–specific FN-deficient mice and mEGFP-FN reporter mice we were able to show that these macrophages were the primary source of FN that helped in the healing process. A final observation worth mentioning is that the FN not only entered the blood stream but was also able to selectively enter the wound site — but not adjacent healthy skin — highlighting the importance of selective increases in microvascular permeability at the afflicted tissue.

LPMs originate from the fetal liver during development before birth and maintain one of the largest populations of macrophages in the body through local environmental factors, including retinoic acid and other unidentified molecules produced in the peritoneum and omentum (9–11, 48, 49). LPMs from different cavities including the peritoneum, pleura, and pericardium exhibit nearly identical genetic profiles based on bulk RNA sequencing and have been shown to contribute to healing in at least the peritoneal and pericardial space (14, 36). Upon peritoneal cavity perturbation, a sharp decline in LPM numbers occurs, a phenomenon known as macrophage disappearance reaction, which has been observed in both vertebrates and invertebrates (15–17, 30, 50–52). Our data suggest that this term may be misleading, as, using a new lineage-tracing mouse, we show that these cells did not leave the peritoneum to be recruited into the blood stream or at the remote site of injury. We also did not see them in the draining lymph nodes, but we did see many of them migrate to the omentum (unpublished data) where they may release FN near the milky spots — key draining structures for the peritoneum. Why LPMs move to milky spots is not clear, but we would postulate that it is here that the LPMs release FN, and perhaps other molecules, for remote healing. To our knowledge, only one publication has suggested that LPMs can enter the bloodstream and lungs following the depletion of alveolar macrophages (19); however, in that study, the LPMs were nonspecifically labeled with nanoparticles, an indirect method that could have been taken up by monocytes that are very capable of replacing alveolar macrophages.

Systemic endocrine responses to local injury are well established in complex organisms. For example, stress hormones during injury trigger the liver to release acute phase proteins (53). Acute phase proteins range from cytokines to complement and coagulation factors but also include extracellular matrix proteins such as FN (54). It is worth noting that, to date, liver hepatocytes were thought to be the dominant source of plasma FN (55). However, a recent publication analyzing macrophages within the peritoneal cavity reported that they are a major source of FN, producing levels that were unexpectedly 25-fold greater than those produced by the liver (22). Although that study relied exclusively on transcriptomic data, our findings corroborate and extend it by showing that LPMs increase FN levels after peritoneal stimulation and indicate that plasma FN is not derived solely from the liver. These levels of FN were sufficient for the early formation of the provisional matrix that is then remodeled by recruited immune cells and fibroblasts (56, 57). The more rapid reepithelialization of skin wounds seen after peritoneal stimulation is consistent with the delivery and bioactivity of additional levels of FN beyond that produced locally at the injury site (58). Exogenous administration of FN has been used to improve wound healing (58, 59), and our data are in line with this work and demonstrate an endogenous central source of FN within the peritoneum, the sphere of influence of which extends well beyond the cavity to remote injury sites.

There is a large body of evidence suggesting that FN is a critical component of the skin wound healing response, having multiple functions (60, 61). As an ECM protein organized into a complex, fibrillar network, it functions as a scaffold for collagens, proteoglycans, and other matrix proteins (60, 62, 63). In addition, while FN is instantly released and deposited by cells locally, it also circulates in the blood to help form the provisional ECM supporting wound healing (43). This systemic source of FN may become important when local cells — and, as such, local sources of FN — are ablated, such as during a burn, at which point only circulating FN is available (64). Notably, excessive FN production could potentially drive fibrosis or excessive scarring; however, we did not observe keloids or hypertrophic scars in our cohort. While we experimentally stimulated the peritoneum for proof of concept that LPMs can perform endocrine functions, to increase healing of small remote skin wounds, it is conceivable that larger wounds as well as systemic trauma would endogenously activate LPMs for increased remote healing.

In conclusion, our data strongly support the view that large peritoneal cavity macrophages, which were found in all metazoan life forms, play critical roles in not just local healing but at remote sites, particularly in complex species with multiple compartments. Indeed, our observations extend beyond mouse models to human peritoneal macrophages, where we observed a high expression of FN and identified that preoperative serum FN was a potential biomarker for predicting postoperative complications in humans. While this was a proof-of-concept study restricted to examining FN, it is possible that the LPMs release other healing factors and — perhaps in diabetic and other chronic skin wounds — activating LPMs (rather than injected FN) could have the capacity to dramatically improve healing responses.

## Methods

### Sex as a biological variable

Both male and female mice were used in this study. Sex was not considered as a biological variable. For human studies, data were collected from both men and women.

### Experimental animals

All mice were maintained on the C57/Bl6J background. Our study examined male and female animals, and similar findings are reported for both sexes. Specific-pathogen-free (SPF) WT C57BL/6J (Jackson Laboratory), *Lyz2^cre^* (Jackson Laboratory), *Gata6^fl/fl^*, *Fn^fl/fl^* (Jackson Laboratory), *C3^–/–^* (Jackson Laboratory), *Fn^gfp/gfp^*, *Ccr2^RFP/RFP^* were bred and housed in individual ventilated cages in the Animal Facility of the University of Bern (Switzerland) or in the Animal Facility of the University of Calgary (Canada). The mice were fed autoclaved rodent feed and water ad libitum. Male and female mice between 8 and 12 weeks were used for experiments.

The *Gata6^fl/fl^* mice were kindly provided by Ruslan Medzhitov (9) (Yale School of Medicine, New Haven, Connecticut, USA) and bred in-house with *Lyz2^cre^* to generate Cre+ (denoted as Mac-Gata6 KO) and Cre- (Mac-Gata6 WT).The generation of *Ccr2^RFP/RFP^* mice (*Ccr2* ^–/–^, Richard Ranshoff, Cleveland Clinic, Ohio, USA) has been previously described (32, 65, 66). Gata6-tracer mouse: The Gata6-tracer mouse model was established by crossing three genetically modified mouse strains. First, a Lysozyme M (*LysM*) promoter-driven Cre recombinase strain was crossed with a tamoxifen-inducible *Gata6-Flippase (Flp,* Cyagen*)* strain, which includes a 2A linker to maintain normal *Gata6* expression. Subsequently, the offspring were crossed with an Ai65 reporter strain (Jackson Laboratory) containing Cre and Flp-sensitive stop sequences upstream of the tdTomato gene (Figure 4A). This model ensures that only cells which have previously expressed Lysozyme M and are actively expressing *Gata6* at the time of tamoxifen administration will permanently express tdTomato, allowing for efficient lineage tracing of LPM populations. Mice were fed a tamoxifen-containing diet for three weeks following weaning, followed by a two-week washout period before further experimentation. The *Fn^fl/fl^* mice were bred with *Lyz2^cre^* mice to generate Cre+ (denoted Mac-Fn KO) and Cre- (Mac-Fn WT). *Fn^gfp/gfp^* mice were kindly provided by Sophie Astrof (Rutgers University, Piscataway, New Jersey, USA).

### Mouse model

In this new mouse model, we combined a sterile peritoneal injury with an additional skin injury. In brief, mice were anesthetized with a mixed triple combination of Fentanyl (0.05mg/ml), Midazolam (5mg/ml) and Medetomidin (1mg/ml). The abdominal area was shaved to remove hair and subsequently disinfected with ethanol to ensure a sterile environment for the procedure. In the peritoneal stimulus group (PS+SW), a 2 cm midline laparotomy was performed and closed in two layers, with separate continuous suturing (Prolene 6-0). Subsequently, a full-thickness excisional skin wound was generated on the right flank using disposable 4mm biopsy punches. The skin wound was intentionally placed on the distant skin away from the peritoneal cavity to enable assessment of the systemic effect on wound repair. In the wound only group (SW) a 4 mm excisional skin wound was performed. Intraperitoneal administration of *E*. *coli*, LPS, and ATP was performed at the time of wounding. After surgery an antidot mix of Buprenorphin (0.1mg/kg), Flumazenil (0.5mg/kg) and Atipamezol (2.5mg/kg) was administrated.

### Parabiosis

Parabiotic pairs were generated as previously described (67). Age-matched female mice were cohoused for three weeks prior to parabiosis surgery to promote social acclimatization. Anesthesia was induced and maintained with isoflurane. Ophthalmic lubricant was applied to prevent corneal drying, and buprenorphine (0.1 mg/kg) and warmed saline were administered subcutaneously for analgesia and perioperative fluid support. A skin incision was made from the shoulder to the hip along the lateral flank of each mouse. The incisions were aligned, and sutures were placed at the shoulder and thigh skin and muscle layers. The remaining incisions were then closed along the skin flaps with a 6-0 monofilament polypropylene running suture (Prolene 6-0).

### Human samples

We obtained blood samples from patients who were undergoing major abdominal surgeries including pancreatic, colorectal and hepatic resections at the Department of Visceral Surgery and Medicine, Inselspital Bern, Switzerland. Postoperative complications were classified according to the Clavien-Dindo grading system (46, 68, 69). For the patient cohort, peripheral blood was collected by venipuncture into EDTA-containing tubes and processed immediately. Platelet-rich plasma was isolated using minimal handling to limit artifactual platelet activation.

### Collection of skin wound, peritoneal fluid, blood and other organs

Prior to harvesting, mice were anesthetized using 2 μl/kg mouse body weight of a mixed triple combination as described above. The skin wounds were excised with a 8mm biopsy disposable punch. To collect the peritoneal fluid, 15ml ice cold PBS were injected intraperitoneally using a 10ml syringe and a 18 gauge plastic catheter. The peritoneal fluid was collected in a 50 ml Falcon tube (70). Tubes were centrifuged at 350 g for 7 min to pellet cells present in the peritoneal fluid. Blood was collected from the inferior vena cava using a 22 gauge plastic catheter. Blood was incubated for 60min at room temperature and centrifuged at 2000*g* for 20 min. Serum was collected in a new Eppendorf tube and frozen for future experiments. Various organs, including liver, lungs, kidney, mesenterial lymph nodes, small intestine, colon and fecal pellets were snap frozen in liquid nitrogen for future experiments.

### Activation of mouse peritoneal macrophages

To collect peritoneal macrophages, the peritoneal cavity was washed with ice cold PBS. After cells were centrifuged at 350*g* and 4°C for 7 minutes and resuspended in HBSS medium. Then, 100uM ATP and 2mM was added to the treatment group and incubated in the cell-culture incubator for 20 minutes in a ultralow adherent 6 well plate (StemCell, # 38071), as described previous (14). The cells were then centrifuged for 350*g* for 7 minutes and the supernatant was collected to measure the fluorescence intensity.

### Flow cytometry

To perform flow cytometry, cells were isolated from the skin wounds and from the peritoneal cavity. Peritoneal cells were isolated by washing the peritoneal cavity with ice cold PBS ^49^. The skin wound was cut in small pieces and placed in 5ml Iscove`s Modified Dulbecco`s Medium (IMDM) containing 2% FBS, 1mg/ml collagenase I and 0,1mg/ml DNase I (Roche) with shaking at 37°C for 90 min. Single cell suspension was passed through 100 μm cell strainer.

After isolation, cells (peritoneal cells or skin wound cells) were washed once with PBS. Cells were processed in 96-well V-bottom plates and all staining steps were performed at 4°C in the dark. Cells were incubated with a fixable viability dye (AmCyan, eBioscience #65-0866-14 or GhostRed710, Tonbiosciences #120871-T100) and Fc receptor block (anti-moues CD16/CD32, BioLegend, San Diego CA) in PBS for 20 minutes at 4°C to exclude dead cells and to minimize nonspecific binding. Single-cell suspensions were incubated with fluorescence-coupled antibodies dilutes in FACS buffer (Supplemental Table 1). Compensation of fluorescence emission was performed using ultra-compensation beads (BD Biosciences). Finally, cells were acquired on LSR Fortessa (BD) or Cytek Aurora and data were analyzed with FlowJo software (Treestar Data Analysis Software) and OMIQ (https://www.omiq.ai/).

### Histology and immunohistochemistry

For histological analysis, sections were consistently taken through the center of the wound to allow a standardized assessment. Fresh skin wound tissue was fixed for 4 hours in 4% paraformaldehyde. For hematoxylin-eosin (H&E) staining, sections were deparaffinized with xylol and then counterstained with H&E (H-51275, E6003; Sigma-Aldrich Chemie GmbH, Steinheim, Germany). For immunohistochemistry, paraformaldehyde-fixed tissue was cut into 3.5μm thick sections followed by deparaffinization, rehydration, and antigen retrieval with citrate buffer. Unspecific binding was blocked with 3% goat serum, 0.5% casein, 0.025% Tween20 and endogenous peroxidase was quenched with 3% H_2_O_2_. Sections were incubated first with anti-F4/80 (invitrogen, MA5-16363) followed by biotinylated anti-rabbit (Dako, BA1000) and ABC-HRP reagent (Vector Laboratories, VC-PK-7100). After incubation of the tissue sections with DAB (Sigma, D3939), slides were counterstained with hematoxylin (H-51275; Sigma-Aldrich Chemie GmbH, Steinheim, Germany). Stained sections were imaged with the Panoramic 250 automated slide scanner (3DHistech version 3.0.2) at 20x magnification. Epithelial gap, epidermal thickness and length of epithelial tongue were quantified using Fiji v.2.16.0 (71).

### IMC

#### IMC tissue staining.

To prepare for IMC, mouse formalin-fixed paraffin embedded (FFPE) tissue was sliced with a microtome into 3.5 μm thick sections and placed on a SuperFrost Plus Adhesion slide (Thermofisher, J1800AMNZ). The tissue was positioned in the middle of the slide to ensure it is in the laser accessible region of the slide. The slide was dried at 37°C overnight and a consecutive slide was stained with H&E to determine the regions of interest (ROI) to acquire on the IMC. Briefly, and as described in detail in OMIP-088 (72), tissue was dewaxed with Xylol and EtOH (Xylol 2 x 10 minutes, 100% EtOH 2 x 5 minutes, 94% EtOH 1 x 5 minutes, 70% EtOH 1 x 5 minute) and then antigen retrieval was performed in citrate buffer at pH6 in the Decloaking Chamber™ NxGen at 110°C for 15 minutes. Slides were then rinsed in Maxpar Water (Standard BioTools, 201069) and blocked in blocking solution (TBS, 0.1% NaN3 (Merck, 106688), 3% Goat serum (Thermofisher, 16210-064), 0.5% Casein (Sigma, C-8654), 0.025% Tween20 (Sigma, P1379)) for 1 hour at room temperature. The slide was then stained overnight at 4°C in antibody cocktail (Supplemental Table 2) and rinsed with DPBS + Tween20 before staining with Cell-ID Intercalator-Ir in DPBS for 30 minutes at room temperature. The intercalator was washed off in Maxpar Water and the slide was dried for a minimum of 20 minutes under an airflow hood.

#### Sample acquisition on imaging mass cytometer.

Images were acquired on a Standard BioTools Hyperion Imaging System with the CyTOF 7 software operated by the IMC Platform at the University of Bern and Inselspital. Quality control is ensured by first running an automated QC program on the Standard BioTools tuning slide that is coated with 3 metal isotopes (^175^Lu, ^89^Y and ^140^Ce). When all parameters are within the specified ranges, we insert the sample slide and align a downloaded tissue image with the camera view image. Once the co-alignment is complete, we take a high-resolution panorama image of the tissue to aid us to locate the ROI selected in the H&E tissue image. The ROIs are then drawn on the panorama image and the channels to open for detection are selected in an acquisition template. We acquired these images at 200 hz ablation frequency and a laser power of 2.

#### Data preprocessing.

Raw imaging mass cytometry (IMC) data files were visualized and checked for image quality in napari v.0.4.17 (73) using napari-imc plugin v0.6.5. (74). Minor acquisitions labelled as lp1, lp2, and lp3 were used for Hyperion laser tests and discarded from further analysis. Remaining regions of interest (ROI) were segmented using napari-steinpose plugin v0.01.1 applying cellpose v2 tissuenet sgmentationmodel with parameters diameter 8.0, *flow_threshold = 1, cellprob_threshold = -3*. Channels DNA1 and DNA2 were used as representing nuclear signal, and channels ICSK1, ICSK2, B220, CD44, CD45, CD11b, CollagenT1, and Vimentin – representing cytoplasmic or membrane signal. Resulting segmentation masks were used as a part of steinbock v0.16.1 pipeline (74). Briefly, raw IMC image data was filtered for hot pixels using *steinbock preprocess imc images --hpf 50* command. Using napari-steinpose-produced masks, cell channel intensities and spatial region properties were measured using *steinbock measure intensities* and *steinbock measure regionprops* commands. 5 nearest neighbor graphs were constructed using *steinbock measure neighbors --type centroids --kmax 5*. Individual channel images for quantification were generated using *steinbock export histocat* function.

#### IMC data analysis.

Resulting datasets were imported into R v.4.3.0 in Rstudio v.2024.9.0 using imcRtools package v.1.7.0 (73). Data was analyzed according to the “IMC Data Analysis Workflow” at https://bodenmillergroup.github.io/IMCDataAnalysis/index.html, without spillover and batch effect correction steps. Prior to any stochastic calculations seed was set to 322. IMC dual counts were arcsinh-transformed and used for dimensionality reduction on individual cell level using scater v.1.28.0 via r*unUMAP* and *runTSNE* functions with default parameters. To minimize false positive segmentation calls, cells were sorted for DNA1 and DNA2 channel content using CATALYST v.1.24.0 (75) function *cluster*; cells with low DNA content were removed. To annotate the remaining cells, consecutive rounds of CATALYST *cluster* applications were performed over informative channels. Markers associated with specific cell types were used to separate CD4^+^, CD8^+^, Tregs, Neutrophils, Macrophages, B cells, and Fibroblasts. Additional cells without specific markers with high expression of Granzyme B and MHCII were classified as such. Stromal cells were separated into skin/muscle cells, endothelial cells, collagen-rich cells and FN-rich cells based on their location and dominant marker expression. Remaining unclassified cells were labelled as undefined. After classification, differential abundance testing was performed over cell types between conditions using edgeR v.3.4.2. (76) as described in chapter 6 of “Multi-Sample Single-Cell Analyses with Bioconductor” at https://bioconductor.org/books/3.13/OSCA.multisample/ (77). Cell counts per donor mouse were pooled together. Collagen and FN intensity per image were quantified using Fiji v.2.16.0 (71)

### Proteomics

#### Shotgun proteomic analysis.

Shotgun proteomics was performed on the previously described flash-frozen supernatant of peritoneal fluid, that was being stored at –80°C. Total protein concentrations for each sample were then determined using a ThermoFisher BCA kit.

As previously described samples were prepared using the filter-assisted separation of peptides (FASP) method. 100 μg of protein was precipitated by adding trichloroacetic acid (TCA) followed by an incubation on ice. Samples were then centrifuged at 14,000*g* for 15 minutes at 4°C, washed 3 times in ice cold acetone, and stored at –20°C. Samples were resuspended in 8M tris-urea solution by shaking and then denatured with the addition of 10 mM DTT at 37°C for 30 minutes. 50 mM iodoacetamide was added in the dark at room temperature to complete carbamidomethyl modification of the cystines. Samples were moved to the top of a 30 kDa filter along with 100 uL of wash solution and were then centrifuged at 14,000 x g for 15 minutes. Samples were then washed 3 times with 8 M tris-urea and 3 times with 50 mM ammonium bicarbonate. The samples were trypsinized at 37°C overnight at a ratio of 1:10 trypsin: total protein, and subsequently, eluted off the filter membrane by washing with 50 mM ammonium bicarbonate. Samples were labeled with light formaldehyde or heavy formaldehyde, 40 mM of formaldehyde was added followed by 20 mM final of sodium cyanoborohydride. The pH was adjusted to 6.5 before incubation at 37 °C for 2 hours. Samples were then mixed in a 1:1 ratio before they were subjected to a c18 clean up with waters solid-phase extraction (SPE) column, according directions from the manufacturer, before injection on the orbitrap mass spectrometer In DDA mode.

#### High performance liquid chromatography (HPLC) and mass spectrometry (MS).

Using a process previously described by Gordon et al., 2019, tryptic peptides were analyzed on an Orbitrap Fusion Lumos Tribrid mass spectrometer (Thermo Scientific) operated with Xcalibur (version 4.4.16.14) and coupled to a Thermo Scientific Easy-nLC (nanoflow Liquid Chromatography) 1200 system. A total mass of 2 μg tryptic peptides were loaded onto a C18 trap (75 um x 2 cm; Acclaim PepMap 100, P/N 164946; ThermoScientific) at a flow rate of 2 μl/min of solvent A (0.1% formic acid in LC-MS grade water). Peptides were eluted using a 120 min gradient from 5 to 40% (5% to 28% in 105 min followed by an increase to 40% B in 15 min) of solvent B (0.1% formic acid in 80% LC-MS grade acetonitrile) at a flow rate of 0.3 μL/min and separated on a C18 analytical column (75 μm x 50 cm; PepMap RSLC C18; P/N ES803; ThermoScientific). Peptides were then electrosprayed using 2.1 kV voltage into the ion transfer tube (300°C) of the Orbitrap Lumos operating in positive mode. The Orbitrap first performed a full MS scan at a resolution of 120000 FWHM to detect the precursor ion having a m/z between 375 and 1575 and a +2 to +7 charge. The Orbitrap AGC (Auto Gain Control) and the maximum injection time were set at 4e5 and 50 ms, respectively. The Orbitrap was operated using the top speed mode with a 3 sec cycle time for precursor selection. The most intense precursor ions presenting a peptidic isotopic profile and having an intensity threshold of at least 5000 were isolated using the quadrupole and fragmented with HCD (30% collision energy) in the ion routing multipole. The fragment ions (MS^2^) were analyzed in the ion trap at a rapid scan rate. The AGC and the maximum injection time were set at 1e4 and 35 ms, respectively, for the ion trap. Dynamic exclusion was enabled for 45 sec to avoid of the acquisition of same precursor ion having a similar m/z (plus or minus 10 ppm).

#### Proteomic data and bioinformatics analysis.

Spectra data obtained during mass-spectrometry were matched to peptide sequences from a Mouse FASTA reference file obtained from Uniprot on Aug 11^th^ 2017 using MaxQuant (v.2.4.9.0). Variable modifications included Oxidation on Methionine, Acetylation on any N-terminus, and Deamidation on Asparagine and Glutamine. Samples were requantified and matched between runs. The first search peptide tolerance was set to 10, the minimum peptide length was set to 5, and the maximum mass was set to 6,600 Da. The minimum score for the modified peptides was set to 20. All other MaxQuant settings were set to default.

MSstatsShiny UI (v0.1.0; https://msstatsshiny.com/app/MSstatsShiny) running MSstats (v.4.2.0) was used to run the statistical analysis. The MaxQuant files “ProteinGroups” and “evidence” were used for the analysis, while the annotation file was created according to directions provided by MSstats. Unique peptides were included in the analysis. For data processing, log_2_ was defined for data transformation and equalize medians was the method chosen for normalization. The options that were enabled for this analysis were “Use all features”, “assume all NA as censored”, “Do not apply cutoff to censor missing values”, and “Model based imputation”. Statistical inference was performed using custom pairwise comparisons, and the data was imported to excel for final assessment. Significant enriched proteins for each group were found using a p-value cut off of 0.05.

### Bulk RNAseq

#### RNAseq data processing.

Data were obtained previously published liver (control samples only) (45) and cavity macrophages (peritoneal macrophages samples only) (36). Reference for alignment was built using salmon (78) v.1.10.0 *index* function using a decoy-aware transcriptome (79). ENSEMBL (80) mouse genome GrCm39 release 110 combined with its transcriptome was used as a transcriptome input and genome itself as a decoy; k-mer size was set to 21. RNAseq studies were aligned to the resulting reference and quantified using salmon *quant* function with --libType A, --validateMappings, and otherwise default parameters.

#### RNAseq data analysis.

Processed data were imported into into R v.4.3.0 in Rstudio v.2024.9.0 using tximport (81) package v.1.32.0 and analyzed using DRIMseq (82) v.1.32.0 Protein encoding FN isoforms were classified as cellular when containing any of the extra domain A (EDA) or extra domain B (EDB), and plasma if both are absent (83).

### Single cell RNA sequencing

#### Single cell analysis.

Single-cell RNA sequencing (scRNA-seq) data were analyzed using the Scanpy pipeline (v1.10.1) (84, 85). To ensure data quality, cells were filtered using predefined thresholds. Cells with fewer than 500 or more than 30,000 detected genes, or with mitochondrial gene content exceeding 20%, were excluded. Genes expressed in fewer than 20 cells were removed, and a minimum threshold of 150 genes per cell was applied.

Raw count data were normalized using `sc.pp.normalize_total` function and subsequently log-transformed. Highly variable genes (HVGs) were identified using `sc.pp.highly_variable_genes`, retaining the top 4,000 HVGs for downstream analyses. Dimensionality reduction and batch correction were performed using scVI, a deep generative modeling framework (86). Two-dimensional visualization was generated using Uniform Manifold Approximation and Projection (UMAP) (87), and clustering was performed with the Leiden algorithm at a resolution of 0.05. Cluster annotation was carried out using a multimodal approach. Differentially expressed genes were identified with `scanpy.tl.rank_genes_groups` (Wilcoxon test) to determine the top marker genes per cluster (Supplemental Figure 7A). Final cluster identities were assigned through manual annotation based on established marker genes from the literature combined with differential expression results.

### FN ELISA

FN levels in human serum were quantified using the Human FN SimpleStep ELISA Kit (ab219046, Abcam). The assay was performed according to the manufacturer’s instructions. Briefly, standards and samples were added to the wells pre-coated with specific antibodies, followed by the addition of a detection antibody. After incubation, the wells were washed, and TMB substrate was added for color development. Absorbance was measured at 450 nm, and FN concentrations were calculated by comparing sample absorbance to the standard curve.

### Compounds

Lipopolysaccharide (LPS) from E. coli O111:B4 (Sigma-Aldrich, L4130; 20 μg in 200 μl) or ATPγS (Jena Bioscience, NU-406–50) was administered as indicated. Clodronate liposomes (Liposoma, C38J0719) and control (PBS) liposomes (Liposoma, P35J0619) were used for macrophage depletion experiments.

### Bacteria

*E. coli* K12 was administered intraperitoneally as described above.

### Statistics

The sample size was determined based on prior experience with similar hypothesis-testing experiments. Data are presented as individual values, with mean ± SD from one representative experiment, unless otherwise specified in the figure legends. Independent experimental repeats produced similar findings. Generally, each data point represents a biological replicate (*n*). The number of independent repetitions of each experiment (*N*) and the number of biological replicates (*n*) are indicated in the figure legends.

Statistical analyses were conducted using GraphPad Prism software or R (RStudio). Two-tailed Student’s *t* tests were used for comparisons between 2 groups. For comparisons of more than 2 groups, ordinary 1-way ANOVA was performed. When ANOVA was applied, multiple comparisons were corrected using Tukey’s test where appropriate. A *P* value of less than 0.05 was considered statistically significant.

### Study approval

Animal experiments were carried out in accordance with Swiss federal regulations and approved by the Animal Care Committee of the Canton Bern, Switzerland (BE16/2022). The experiments conducted in Canada were approved by the institutional animal care committee of the University of Calgary, Alberta, Canada (AC 19-0128, AC 23-0166) and were in compliance with the guidelines from the Canadian Council for Animal Care. The human cohort study was approved by the the Cantonal Ethics Committee of Bern, Switzerland (KEK 2017-00573).

### Data and material availability

All data are available upon request from the corresponding author. The bulk RNA sequencing dataset analyzed in this study was previously published by Deniset et al. (36) and is available in the Gene Expression Omnibus under accession number GEO: GSE131724. The human single cell sequencing dataset of peritoneal cells analyzed in this study was previously published by Han et al. (13) and is publicly available under accession number: GSE228030. The mouse single cell sequencing dataset of peritoneal cells analyzed in this study was previously published by Han et al. (13) and is publicly available under accession number: GSE225668. RAW data were deposited to ProteomeXchange via the Proteomics Identification Database (PRIDE) under accession number (PXD066819). Raw and processed IMC datasets are available at 10.5281/zenodo.18775203. Values for all data points in graphs are reported in the Supporting Data Values file. All reagents and materials used in this study are commercially available. Any request for further information should be directed to and will be fulfilled by the corresponding author.

## Author contributions

Conceptualization: LS, PK, and GB. Methodology: LS, SNZ, D Spari, TY, MS, FVSC, and D Stroka. Formal analysis: LS and TY. Investigation: LS, SNZ, PK, and GB. Resources: GB, PK, AD, and D Stroka. Writing (first draft): LS, PK, and GB. Writing (reviewing): LS, SNZ, D Stroka, TY, MS, FVSC, J Zbinden, D Spari, J Zindel, AD, PK, and GB. Supervision: PK and GB. Funding acquisition: PK and GB.

## Conflict of interest

The authors have declared that no conflict of interest exists.

## Funding support

- Swiss National Science Foundation (SNSF) (#P500PM_203210 and #P5R5PM_225287 to LS, #310030_166594/1 to GB, #TMSGI3_218347 to J Zindel).
- Natural Sciences and Engineering Research Counsil of Canada (NSERC) Discover grant (#RGPIN/07191- 1013 2019 to PK).
- SF Board call University of Bern (to GB).
- Heart&Stroke Foundation of Canada, CIHR and Canadian Excellence Research Chairs (CERC) to PK.

## Supplementary Material

Supplemental data

Supporting data values

## Figures and Tables

**Table 1:**
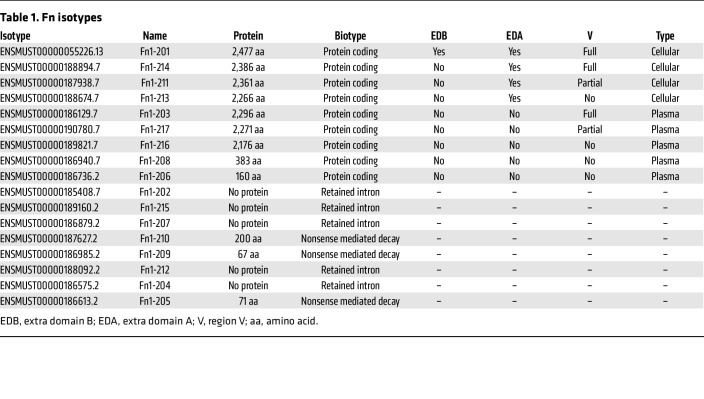
Fn isotypes

## Version 1 03/23/2026

In-Press Preview

## Version 2 05/01/2026

Electronic publication
