# Supplementary material for: Peritoneal macrophages regulate distal wound healing via endocrine release of plasma fibronectin
Source: J Clin Invest. 2026 Mar 12;136(9):e198632. doi: 10.1172/JCI198632 (PMC13132365; doi:10.1172/JCI198632)
Supplement: Supplemental data [file jci-136-198632-s290.pdf]

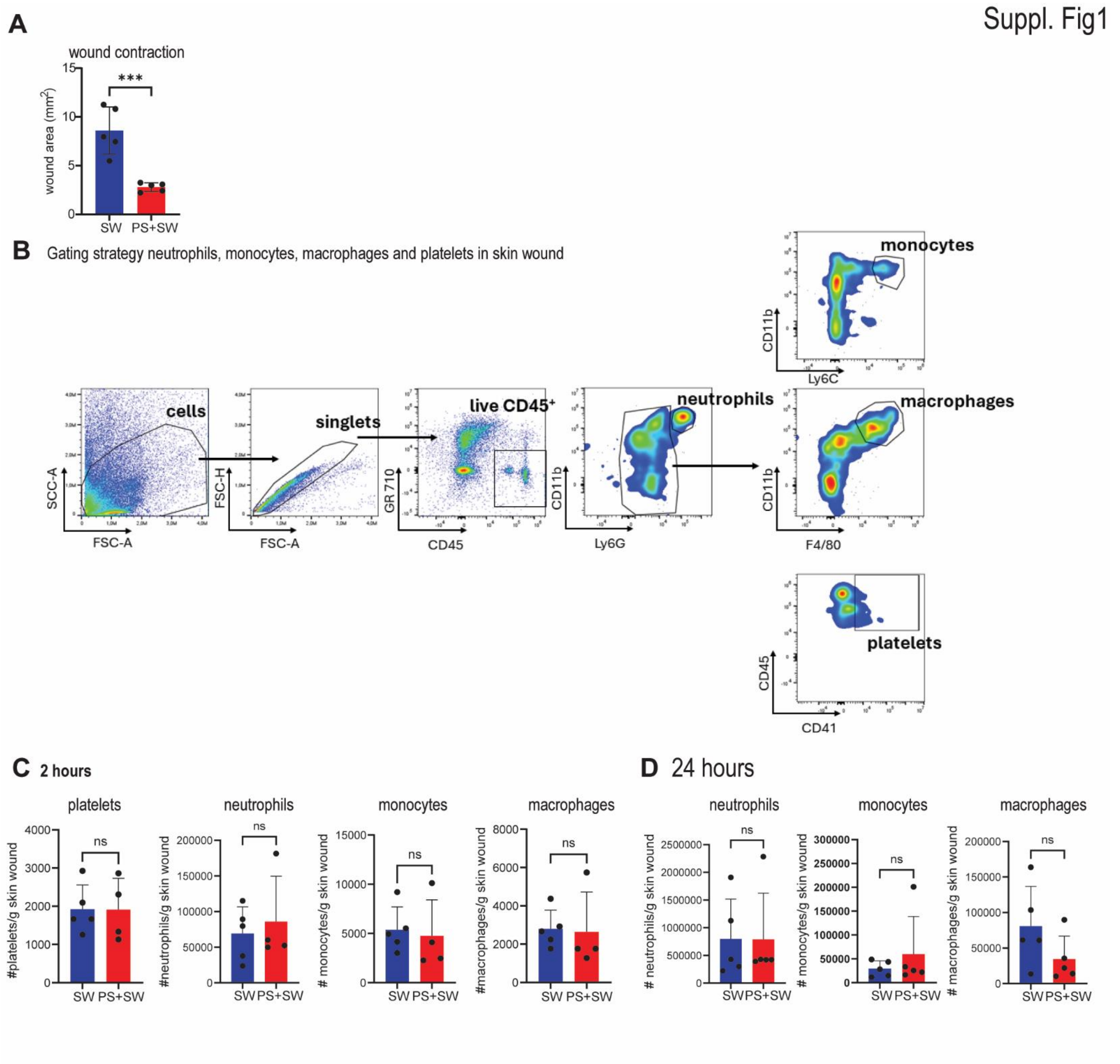
Suppl. Fig1
A
wound contraction
wound area (mm2)
***
SW
PS+SW
B
Gating strategy neutrophils, monocytes, macrophages and platelets in skin wound
cells
singlets
live CD45+
neutrophils
monocytes
macrophages
platelets
SCC-A
FSC-A
FSC-H
GR 710
CD45
CD11b
Ly6G
Ly6C
F4/80
CD41
C 2 hours
platelets
neutrophils
monocytes
macrophages
#platelets/g skin wound
#neutrophils/g skin wound
monocytes/g skin wound
#macrophages/g skin wound
ns
D 24 hours
neutrophils/g skin wound
monocytes/g skin wound
macrophages/g skin wound

1129

Supplemental Figure 1:

(A) Quantification of wound contraction from photographs, n=5 (both groups), p=0.0007, (Students t-test), data denote single mice representative from 1 experiment (N=1). **(B)** Flow cytometry gating strategy of skin wound immune cell populations. **(C)** Quantification of platelets, neutrophils, monocytes and macrophages at 2 hours in the skin wound. N=2 Platelets: p= 0.9838, neutrophils: p= 0.6345, monocytes: p= 0.7624, macrophages: p= 0.8852, (Students t-test) **(D)** Quantification of neutrophils, monocytes and macrophages at 24 hours in the skin wound. N=3, Neutrophils: p= 0.9885, monocytes: p= 0.7397, macrophages: p= 0.1493, (Students t-test)

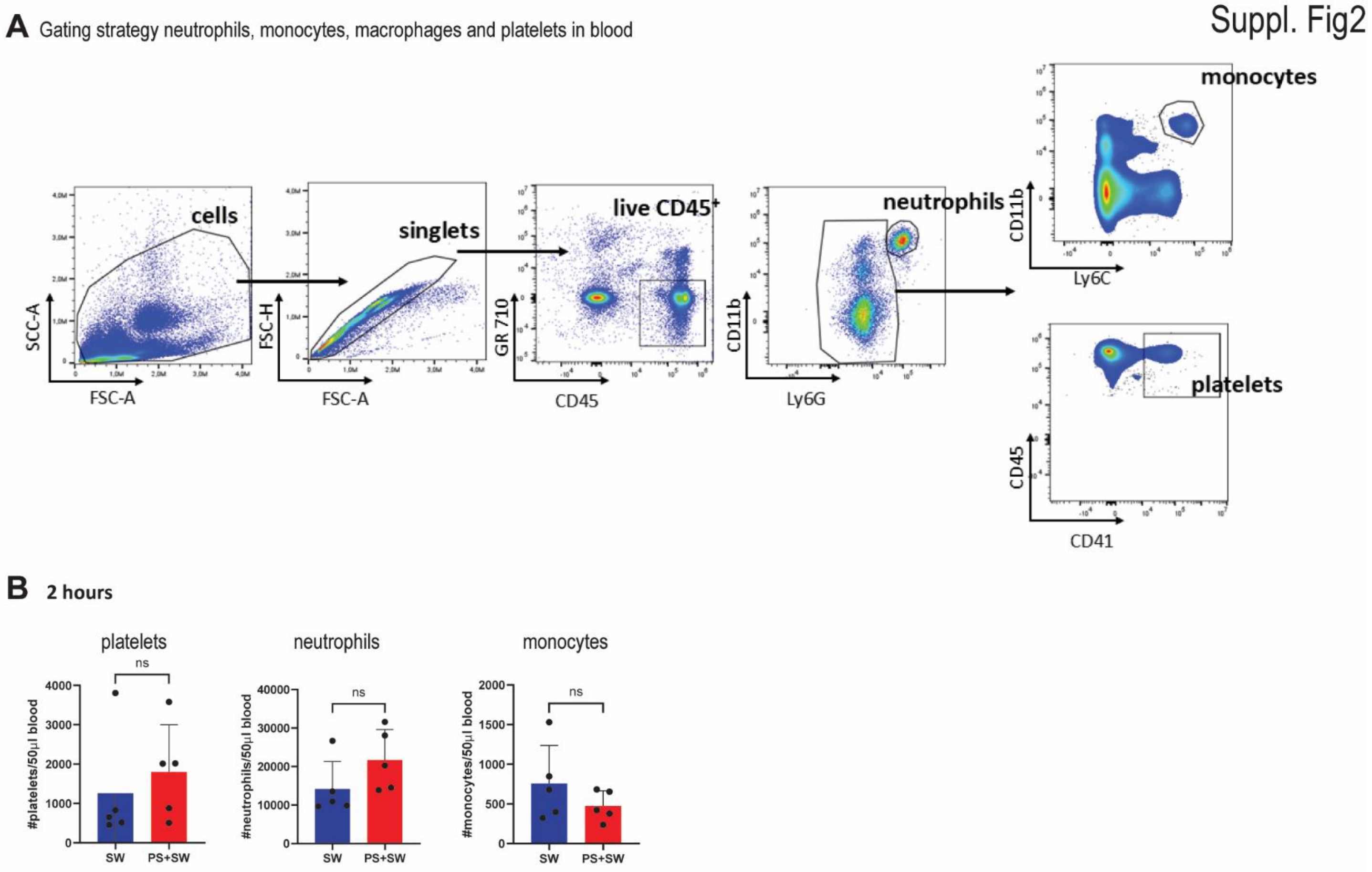

Suppl. Fig2
A Gating strategy neutrophils, monocytes, macrophages and platelets in blood
cells
singlets
live CD45+
neutrophils
monocytes
platelets
SCC-A
FSC-A
FSC-H
GR 710
CD45
CD11b
Ly6G
Ly6C
CD41
B 2 hours
platelets
neutrophils
monocytes
ns
#platelets/50µl blood
#neutrophils/50µl blood
#monocytes/50µl blood
SW
PS+SW

Suppl. Figure 2:

(A) Flow cytometry gating strategy of blood immune cell populations. **(B)** Quantification of platelets, neutrophils and monocytes at 2 hours in the blood. N=2, Platelets: p= 0.5309, neutrophils: p= 0.1553, monocytes: p= 0.2590, (Students t-test)

Each data point represents an individual mouse from one representative experiment. Independent experimental repetitions yielded similar results and are representative of N ≥ 2 experiments.

Suppl. Fig3

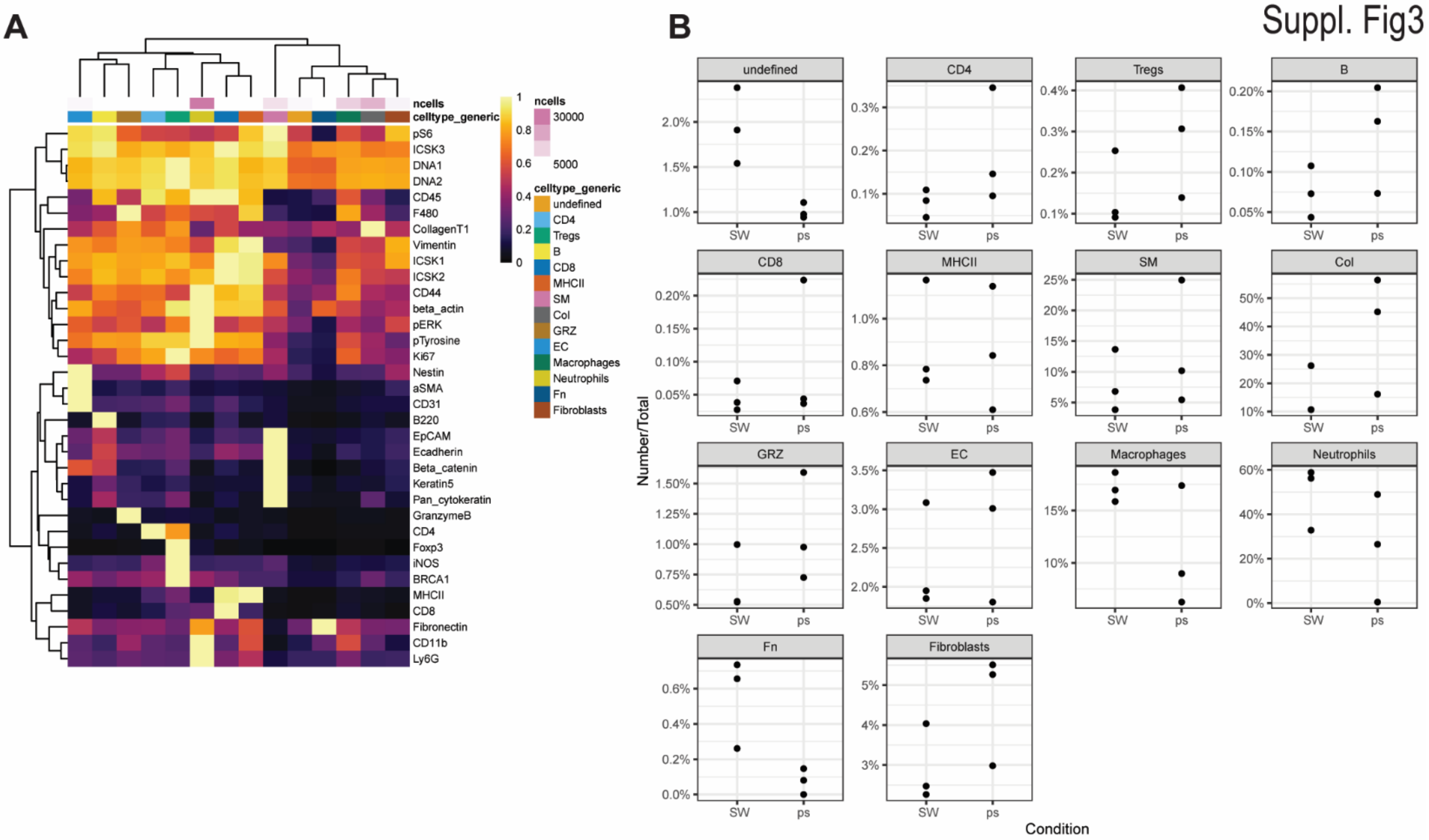

A
B
ncells
celltype_generic
30000
5000
undefined
CD4
Tregs
B
CD8
MHCII
SM
Col
GRZ
EC
Macrophages
Neutrophils
Fn
Fibroblasts
pS6
ICSK3
DNA1
DNA2
CD45
F480
CollagenT1
Vimentin
ICSK1
ICSK2
CD44
beta_actin
pERK
pTyrosine
Ki67
Nestin
aSMA
CD31
B220
EpCAM
Ecadherin
Beta_catenin
Keratin5
Pan_cytokeratin
GranzymeB
CD4
Foxp3
iNOS
BRCA1
MHCII
CD8
Fibronectin
CD11b
Ly6G
Number/Total
SW
ps
Condition

Supplemental Figure 3:

(A) Heatmap showing surface marker expression (y-Axis), which was used to characterize the different immune cell populations (x-Axis). **(B)** Datapoints relative abundance of individual cell types

1

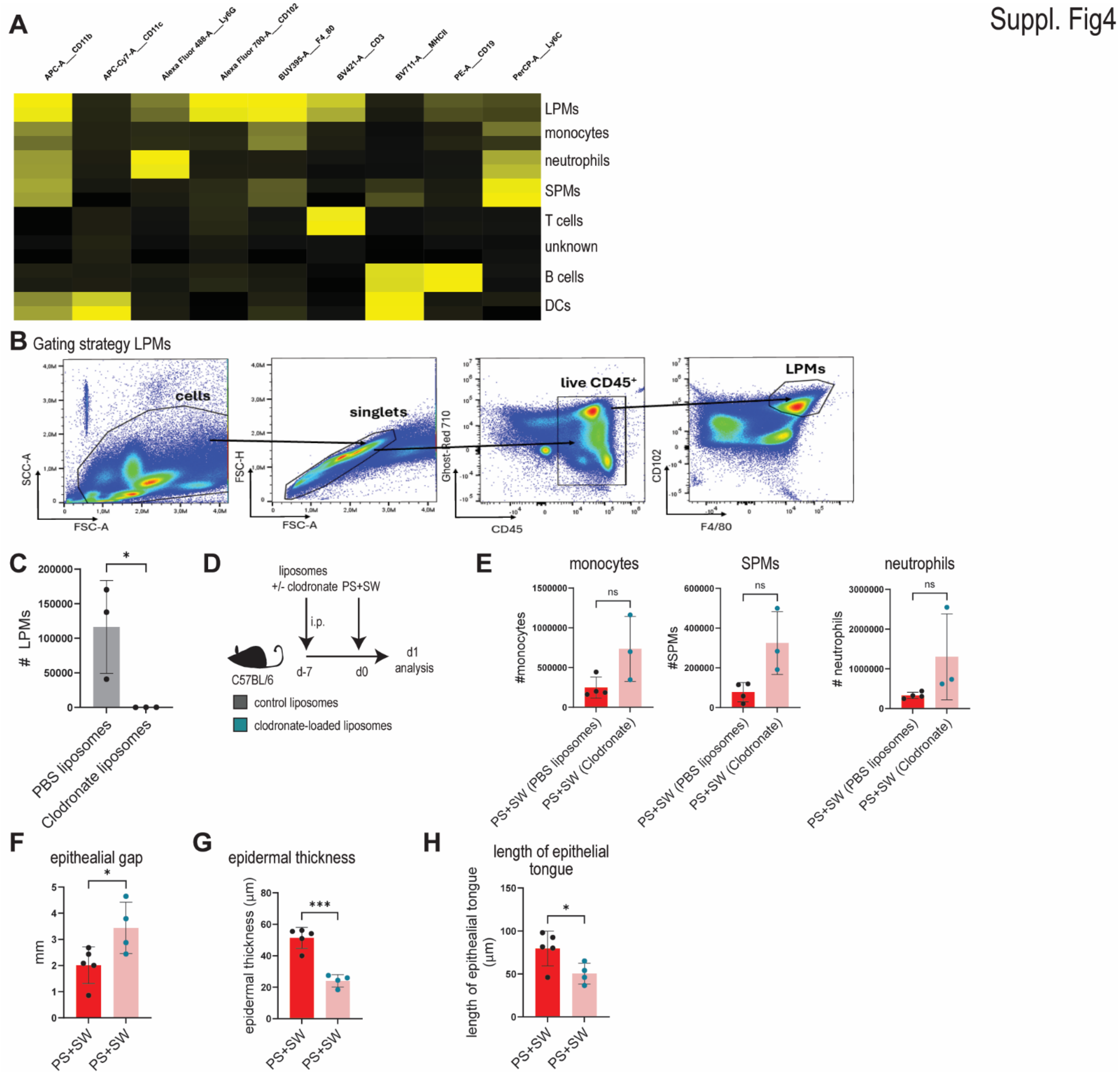
A
APC-A__CD11b
APC-Cy7-A__CD11c
Alexa Fluor 488-A__Ly6G
Alexa Fluor 700-A__CD102
BUV395-A__F4_80
BV421-A__CD3
BV711-A__MHCII
PE-A__CD19
PerCP-A__Ly6C
LPMs
monocytes
neutrophils
SPMs
T cells
unknown
B cells
DCs
B Gating strategy LPMs
cells
singlets
live CD45+
LPMs
SSC-A
FSC-A
FSC-H
Ghost-Red 710
CD45
CD102
F4/80
C
LPMs
PBS liposomes
Clodronate liposomes
D
liposomes
+/- clodronate PS+SW
i.p.
d-7
d0
d1
analysis
C57BL/6
control liposomes
clodronate-loaded liposomes
E
monocytes
SPMs
neutrophils
#monocytes
#SPMs
neutrophils
ns
PS+SW (PBS liposomes)
PS+SW (Clodronate)
F
epitheälial gap
mm
G
epidermal thickness
epidermal thickness (μm)
H
length of epithelial tongue
length of epithealial tongue (μm)
PS+SW

Supplemental Figure 4

(A) Heatmap showing surface marker expression (x-Axis), which was used to characterize the different immune cell populations (y-Axis). **(B)** Gating strategy of LPMs**.** LPMs were defined as singlets, live CD45^{+}, F4/80^{high} and CD102^{+}. **(C)** Quantification of LPMs 7 days after Clodronate liposome ip injection, (PBS liposomes: n=3, Clodronate liposomes: n=3), N=2, p= 0.0404, (Students t-test) **(D)** Illustration of experimental approach **(E)** Quantification of monocytes, SPMs and neutrophils in the peritoneal fluid at day 1. Monocytes: p= 0.1143, SPMs: p=0.0571, neutrophils: p=0.0571. N=2 (Mann-Whitney test). **(F)** Quantification of epithelial gap at day 1, n=5 (PS+SW, control liposomes), n=4 (PS+SW, clodronate-loaded liposomes): p=0.0378, N=1, (Student`s t-test), **(G)** Quantification of epidermal at day 1, n=5 (PS+SW, control liposomes), n=4 (PS+SW, clodronate-loaded liposomes): p=0.0002 (Student`s t-test), N=1 **(H)** Quantification of length of epithelial tongue, n=5 (PS+SW, control liposomes), n=4, N=1 (PS+SW, clodronate-loaded liposomes): p=0.0393 (Student`s t-test).

1 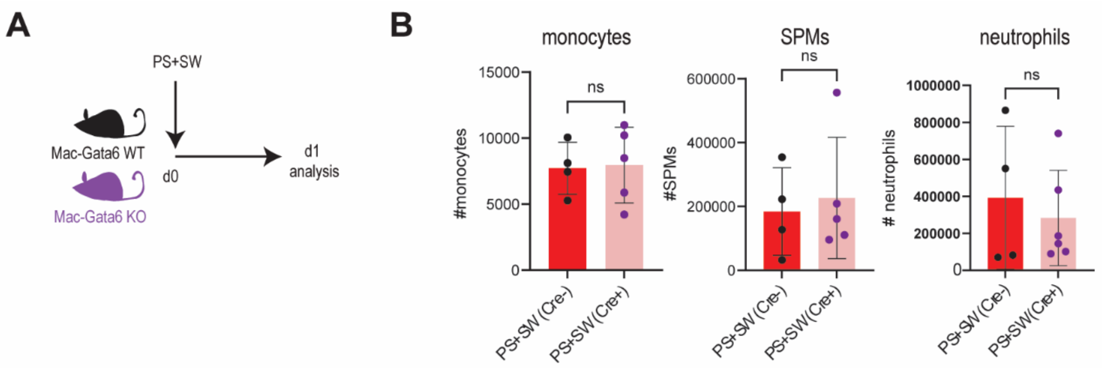
A
PS+SW
Mac-Gata6 WT
Mac-Gata6 KO
d0
d1
analysis
B
monocytes
SPMs
neutrophils
ns
#monocytes
#SPMs
neutrophils
PS+SW (Cre-)
PS+SW(Cre+)

Suppl. Fig5

Supplemental Figure 5

(A) Illustration of experimental approach **(B)** Quantification of monocytes, SPMs and neutrophils in the peritoneal fluid at day1 in wild type and Mac-Gata 6KO mice (PS+SW), monocytes: p=0.8937, SPMs: p=07197, neutrophils: p=0.6018, N=1, (multiple Student`s t-test).

Suppl. Fig6

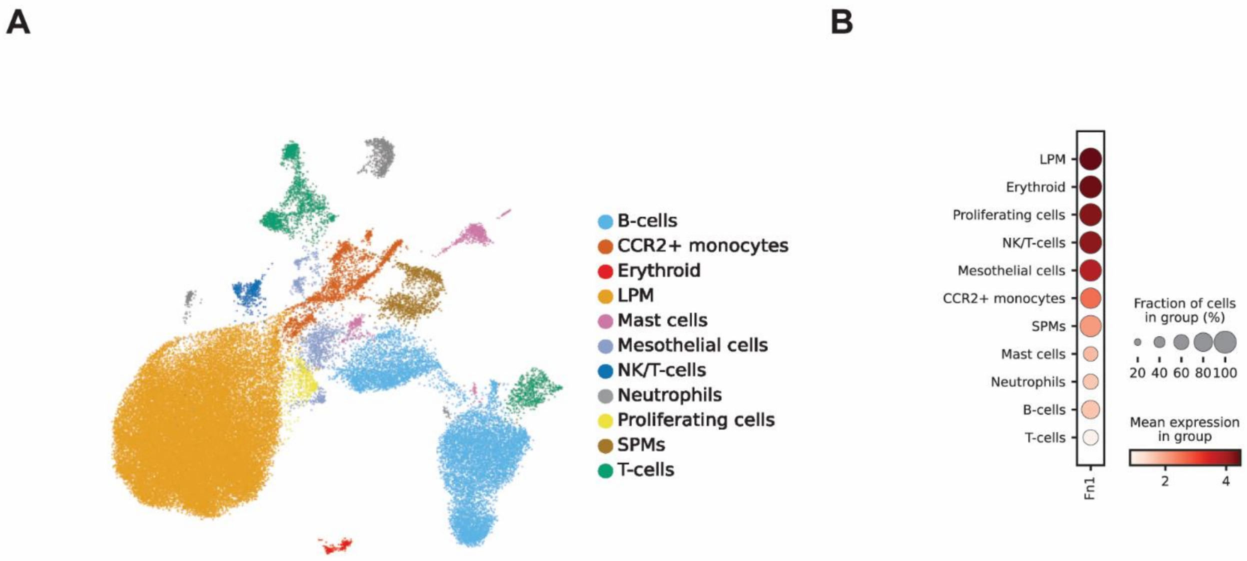
A
B-cells
CCR2+ monocytes
Erythroid
LPM
Mast cells
Mesothelial cells
NK/T-cells
Neutrophils
Proliferating cells
SPMs
T-cells
B
LPM
Erythroid
Proliferating cells
NK/T-cells
Mesothelial cells
CCR2+ monocytes
SPMs
Mast cells
Neutrophils
B-cells
T-cells
Fn1
Fraction of cells in group (%)
20 40 60 80100
Mean expression in group
2
4

Supplemental Figure 6:

(A-B) Supplemental Figure 6A-B were generated using publicly available single cell sequencing data from mouse peritoneal cells from the following source: https://artyomovlab.wustl.edu/scn/?token=gjrandolph.fig1.adult_peritoneal_mnp. **(A)** UMAP-plot of mice peritoneal cells and **(B)** Dot plots showing the average expression levels per cell type of *Fn1*

A Suppl. Fig7

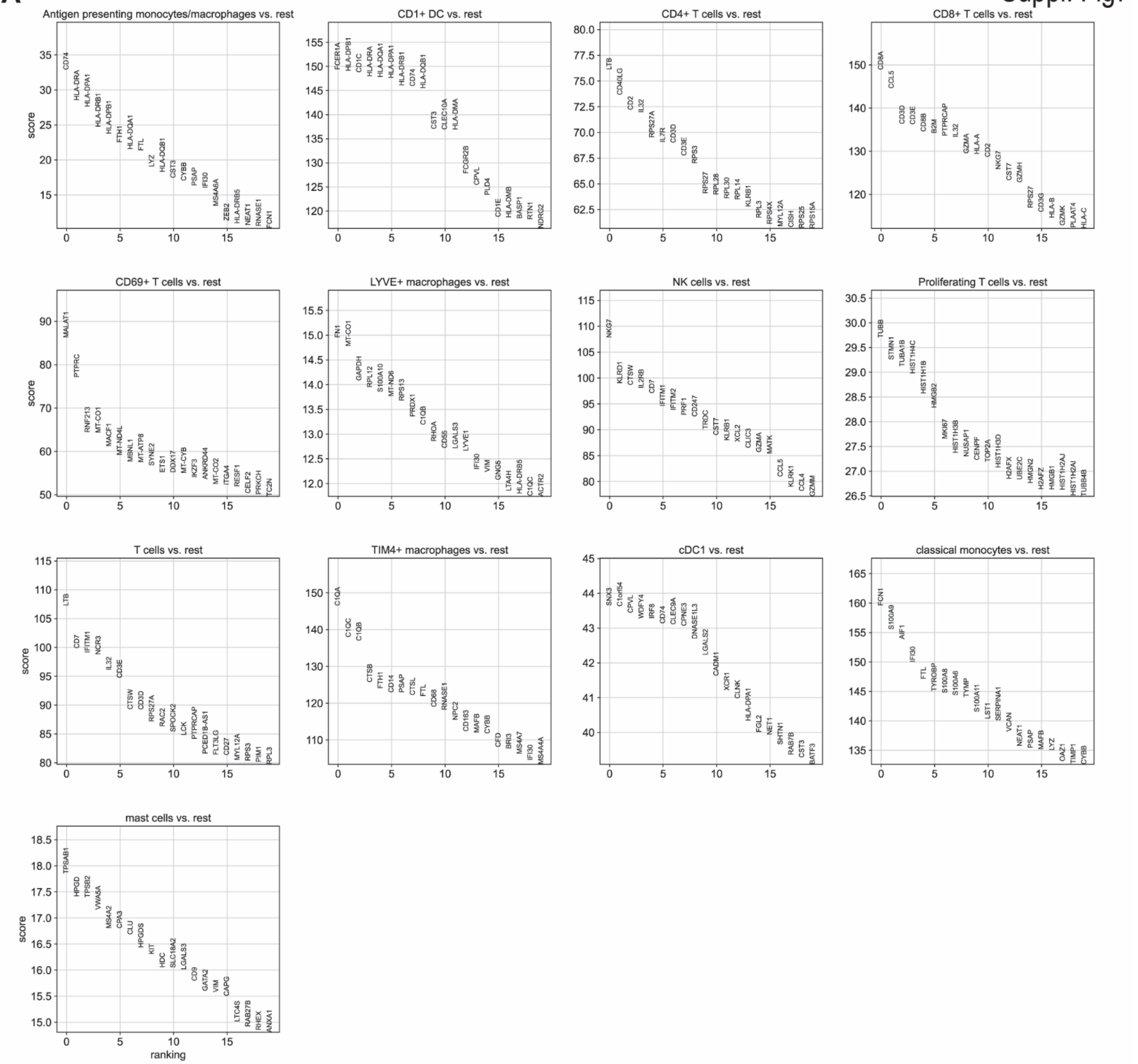

Antigen presenting monocytes/macrophages vs. rest
CD74 HLA-DRA HLA-DPA1 HLA-DRB1 HLA-DPB1 FTH1 HLA-DQA1 FTL LYZ HLA-DQB1 CST3 CYBB PSAP IFI30 MS4A6A ZEB2 HLA-DRB5 NEAT1 RNASE1 FCN1
CD1+ DC vs. rest
FCER1A HLA-DPB1 CD1C HLA-DRA HLA-DQA1 HLA-DPA1 HLA-DRB1 CD74 HLA-DQB1 CST3 CLEC10A HLA-DMA FCGR2B CPVL PLD4 CD1E HLA-DMB BASP1 RTN1 NDRG2
CD4+ T cells vs. rest
LTB CD40LG CD2 IL32 RPS27A IL7R CD3D CD3E RPS3 RPS27 RPL28 RPL30 RPL14 KLRB1 RPL3 RPS4X MYL12A CISH RPS25 RPS15A
CD8+ T cells vs. rest
CD8A CCL5 CD3D CD3E CD8B B2M PTPRCAP IL32 GZMA HLA-A CD2 NKG7 CST7 GZMH RPS27 CD3G HLA-B GZMK PLAAT4 HLA-C
CD69+ T cells vs. rest
MALAT1 PTPRC RNF213 MT-CO1 MACF1 MT-ND4L MBNL1 MT-ATP8 SYNE2 ETS1 DDX17 MT-CYB IKZF3 ANKRD44 MT-CO2 ITGA4 RESF1 CELF2 PRKCH TC2N
LYVE+ macrophages vs. rest
FN1 MT-CO1 GAPDH RPL12 S100A10 MT-ND6 RPS13 PRDX1 C1QB RHOA CD55 LGALS3 LYVE1 IFI30 VIM GNG5 LTA4H HLA-DRB5 C1QC ACTR2
NK cells vs. rest
NKG7 KLRD1 CTSW IL2RB CD7 IFITM1 IFITM2 PRF1 CD247 TRDC CST7 KLRB1 XCL2 CLIC3 GZMA MATK CCL5 KLRK1 CCL4 GZMM
Proliferating T cells vs. rest
TUBB STMN1 TUBA1B HIST1H4C HIST1H1B HMGB2 MKI67 HIST1H3B NUSAP1 CENPF TOP2A HIST1H3D H2AFX UBE2C HMGN2 H2AFZ HMGB1 HIST1H2AJ HIST1H2AI TUBB4B
T cells vs. rest
LTB CD7 IFITM1 NCR3 IL32 CD3E CTSW CD3D RPS27A RAC2 SPOCK2 LCK PTPRCAP PCED1B-AS1 FLT3LG CD27 MYL12A RPS3 PIM1 RPL3
TIM4+ macrophages vs. rest
C1QA C1QC C1QB CTSB FTH1 CD14 PSAP CTSL FTL CD68 RNASE1 NPC2 CD163 MAFB CYBB CFD BRI3 MS4A7 IFI30 MS4A4A
cDC1 vs. rest
SNX3 C1orf54 CPVL WDFY4 IRF8 CD74 CLEC9A CPNE3 DNASE1L3 LGALS2 CADM1 XCR1 CLNK HLA-DPA1 FGL2 NET1 SHTN1 RAB7B CST3 BATF3
classical monocytes vs. rest
FCN1 S100A9 AIF1 IFI30 FTL TYROBP S100A8 S100A6 TYMP S100A11 LST1 SERPINA1 VCAN NEAT1 PSAP MAFB LYZ OAZ1 TIMP1 CYBB
mast cells vs. rest
TPSAB1 HPGD TPSB2 VWA5A MS4A2 CPA3 CLU HPGDS KIT HDC SLC18A2 LGALS3 CD9 GATA2 VIM CAPG LTC4S RAB27B RHEX ANXA1
score
ranking

Supplemental Figure 7

scRNA-seq analysis of human peritoneal cells **(A)** Visualization of each identified cell type cluster represented by individual squares. Each square displays the top 20 genes based on differential expressions within the cell type cluster. Genes are ranked according to expression levels.

Supplemental table 1: Flow cytometry antibodies

Antibody	Source of reference	Identifiers	Dilution
Purified anti-Ms CD16/32, monocolonal	Biolegend	Cat# 101302; clone 93; Lot#B298973; RRID: AB_312801	(1:200)
Fixable Viability Dye eFluor506	Thermo Fisher Scientific	Cat# 65- 0866- 18	(1:600)
GhostRed 710 (AF700)	Tonbiosciences	Cat# 130871-T100, Lot # DO871052323133	(1:4000)
Anti-CD11b (APC)	Biolegend	Cat# 101212; clone M1/70; Lot#B312600; RRID: AB_312795	(1:600)
Anti-CD11c (APC-Cy7)	Thermo Fisher Scientific	Cat# 47- 0114- 80; cloneN418; Lot# 2133269; RRID:AB_1548652	(1:300)
Anti-Ly6G (FITC)	BD Biosciences	Cat# 551460; clone 1A8; Lot#9068981; RRID: AB_394207	(1:100)
Anti-CD102 (Alexa Fluor 700)	Southern Biotech	Cat# 1925-27; clone 3C4; Lot#F1912-SK45B; RRID: AB_2795545	(1:300)
Anti-F4/80 (BUV 395)	BD Biosciences	Cat# 565614; clone T45-2342; Lot# 1104580; RRID:AB_2739304	(1:150)
Anti-CD3 (BV421)	invitrogen	Cat# 48003282; clone 17A2, Lot# 2264580; RRID: AB_2536579	(1:100)
Anti-I-A/I-E (BV 711)	Biolegend	Cat#107643; cloneM5/114.15.2; Lot# B299330;RRID: AB_2565976	(1:600)
Anti-CD19 (PE)	eBioscience	Cat# 12-9668-82; clone: 1A8; Lot#4346371; RRID: AB_657659	(1:800)
Anti-Ly6C (Percp Cy5.5)	eBioscience	Cat#45-5932-82, clone: HK1.4 Lot#2309273; RRID: AB_2723343	(1:100)
Anti-CD45 (AF594)	Biolegend	Cat#103144; clone: 30_F11; Lot#B262565, RRID: AB_2563458	(1:200)
Anti-CD45 (BV 510)	Biolegend	Cat#103138; clone: 30-F11, RRID: AB_2561392, Lot# B386738	(1:200)
Anti-F4/80 (BUV 395)	Biolegend	Cat#123149, clone: BM8, RRID: AB-2564589, Lot# B372797	(1:80)

Anti-CD 102 (BV605)	BD Biosciences	Cat#740346, clone: 3C4(mIC2/4), RRID: AB_2740079, Lot# 3280219	(1:200)
Anti-CD 11b (BV750)	Biolegend	Cat#101267, clone: M1/70, RRID: AB_2810328, Lot# B365976	(1:400)
Anti-CD44 (BUV 395)	BD Biosciences	Cat# 740215, clone: IM7, RRID: AB_2739963, Lot# 2320602	(1:200)
Anti-CD80 (BV 421)	Biolegend	Cat# 104726, clone: 16-10A1, RRID: AB_10900989, Lot# B264062	(1:100)
Anti-CD86 (BUV 615)	BD Biosciences	Cat# 751557, clone: GL1, RRID: AB_2875552, Lot# 3009653	(1:200)
Anti-CD 62p (FITC)	BD Biosciences	Cat# 553744, clone: RB40.34, RRID: AB_395026, Lot# 6140990	(1:100)
Anti-Msr1 (PerCP-Cy5.5)	Biolegend	Cat# 154716, clone: 1F8C33, RRID: AB_2892313, Lot# B333672	(1:100)
Anti-Marco (APC)	R&D systems	Cat# FAB2956A, clone: 579511, Lot# ABHO0420031	(1:100)
Anti-fibronectin (AF647)	abcam	Cat# AB237287, clone: EPR19241-46, Lot# 1022070-1	(1:200)
Anti-Tim4 (BUV 660)	BD Biosciences	Cat# 750205, clone: 21H12, RRID: AB_2874406, Lot# 3299774	(1:100)
Anti-CD36 (PE)	Biolegend	Cat# 102606, clone: HM36, RRID: AB_389348, Lot# B314216	(1:100)
Anti-MerTK (PE dazzle 594)	Biolegend	Cat# 151524, clone: 2B10C42, RRID: AB_2876509, Lot# B364702	(1:400)
Anti-CD206 (PE/Fire 700)	Biolegend	Cat# 141741, clone: C068C2, RRID: AB_2922468, Lot# B402656	(1:80)
Anti-Ly6G (BV650)	BD Biosciences	Cat# 740554, clone: 1A8, RRID: AB_2740255, Lot# 3220031	(1:80)
Anti-Ly6C(Percp)	Biolegend	Cat# 128028, clone: HK1.4, RRID: AB_10900235, Lot# B401995	(1:320)
Anti-F4/80 (PE-CF594)	BD Biosciences	Cat# 565613, clone: T45-2342, RRID: AB_2734770, Lot# 1165073	(1:200)
Anti-CD41 (PE)	BD Biosciences	Cat# 558040, clone: MWReg30, RRID: AB_397004, Lot# 6168978	(1:100)

Anti-CD45 (BV421)	Biolegend	Cat# 103134, clone: 30-F11, RRID: 103134, Lot# B383535	(1:160)

Supplemental table 2: IMC antibodies

Antibody	Clone	Source of reference	Metal Tag	Catalogue number	Lot Number	Dilution
Anti-aSMA	1A4	Standard BioTools	141Pr	3141017D	NA	(1:50)
Anti-Nestin	EPR22023	abcam	143Nd	ab237036	#2209186-12	(1:50)
Anti-pTyrosine	P-Tyr-100	Standard BioTools	144Nd	3144024D	#0461804	(1:100)
Anti-Keratin 5	Poly19055	Biolegend	146Nd	905504	#2209163-09	(1:100)
Anti-EpCAM	EPR20532-222	Standard BioTools	147Sm	91H024147	#2112506-23	(1:100)
Anti-Vimentin	D21H3	Standard BioTools	149Sm	91H049149	#2209091-07	(1:100)
KI67	B56	Standard BioTools	150Nd	91H017150	#24084328-07	(1:100)
Anti-CD45	D3F8Q	Standard BioTools	151Eu	91H029151	#2209084-07	(1:100)
Anti-Fibronectin	EPR19241-46	Standard BioTools	152Sm	91H028152	#2201759-24	(1:100)
Anti-CD44	IM7	Standard BioTools	153Eu	3153029D	#2209184-12	(1:200)
Anti-Beta-actin	2F1-1	Standard BioTools	154Sm	3154021D	#2209352-21	(1:200)
Anti-Granzyme B	EPR22645-206	Standard BioTools	155Gd	91H026155	#2209083-07	(1:200)
Anti-F4/80	D2S9R	Standard BioTools	156Gd	91H030156	#24042373-22	(1:200)

Anti-E-cadherin	24 E 10	Standard BioTools	158Gd	3158029D	#23115540-27	(1:200)
Anti-CD4	BLR167J	Standard BioTools	159Tb	91H031159	#24042446-24	(1:200)
Anti-iNOS	BLR167J	Standard BioTools	160Gd	91H025160	#2209085-07	(1:200)
Anti-MHC class2	M5/114.15.2	Standard BioTools	161Dy	91H038161	#39086-07	(1:400)
Anti-CD8	EPR21769	Standard BioTools	162Dy	91H023162	#2203629-16	(1:400)
Anti-CD11b	EPR1344	Standard BioTools	163Dy	91H007163	#2209087-07	(1:400)
Anti-pERK1/2	D13.14.4E	Standard BioTools	164Dy	91H039164	#2209098-07	(1:400)
Anti-FoxP3	FJK-16s	Standard BioTools	165Ho	91H032165	#2209183-12	(1:400)
Anti-Ly-6G	1A8	Standard BioTools	166Er	91H037166	NA	(1:400)
Anti-CD3e	APA1/1	BioLegend	167Er	362702	#2209095-07	(1:400)
Anti-Beta-catenin	5H10	Standard BioTools	169Tm	91H022169	NA	(1:400)
Anti-CD31	EPR17259	Standard BioTools	171Yb	91H027171	#3031904	(1:500)
Anti-BRCA1	MS110	Standard BioTools	172Yb	3172030D	#2209179-12	(1:500)
Anti-Collagen type 1	NA	Standard BioTools	173Yb	91H018173	#2209094-07	(1:800)
Anti-Pan-cytokeratin	AE-1/AE-3	Standard BioTools	174Yb	91H006174	#1112004	(1:800)
Anti-pS6(s235/s236)	N7-548	Standard BioTools	175lu	3175031D	#22022353-28	(1:1000)

Anti-CD45R/B220	RA36B2	Standard BioTools	176Yb	91H036176	#2209080-07	(1:1000)
Anti-ICSK1	ICSK1	Standard BioTools	195Pt	201500	#2209165-09	(1:1000)
Anti-ICSK2	ICSK2	Standard BioTools	196Pt	201500	#2209078-07	(1:1000)
Anti-ICSK3	ICSK3	Standard BioTools	198Pt	201500	#2211343-30	(1:2000)
